# Babesiosis in Southeastern, Central and Northeastern Europe: An Emerging and Re-Emerging Tick-Borne Disease of Humans and Animals

**DOI:** 10.3390/microorganisms10050945

**Published:** 2022-04-30

**Authors:** Anna Bajer, Ana Beck, Relja Beck, Jerzy M. Behnke, Dorota Dwużnik-Szarek, Ramon M. Eichenberger, Róbert Farkas, Hans-Peter Fuehrer, Mike Heddergott, Pikka Jokelainen, Michael Leschnik, Valentina Oborina, Algimantas Paulauskas, Jana Radzijevskaja, Renate Ranka, Manuela Schnyder, Andrea Springer, Christina Strube, Katarzyna Tolkacz, Julia Walochnik

**Affiliations:** 1 Department of Eco-Epidemiology of Parasitic Diseases, Faculty of Biology, Institute of Developmental Biology and Biomedical Sciences, University of Warsaw, Miecznikowa 1, 02-096 Warsaw, Poland; dorota.dwuznik@biol.uw.edu.pl (D.D.-S.); k.tolkacz@ibb.waw.pl (K.T.); 2 Ribnjak 8, 10 000 Zagreb, Croatia; abeck.cro@gmail.com; 3 Department for Bacteriology and Parasitology, Croatian Veterinary Institute, Savska Cesta 143, 10 000 Zagreb, Croatia; relja.beck@gmail.com; 4 School of Life Sciences, University of Nottingham, University Park, Nottingham NG7 2RD, UK; jerzy.behnke@nottingham.ac.uk; 5 Vetsuisse Faculty, Institute of Parasitology, University of Zurich, 8057 Zürich, Switzerland; ramon.eichenberger@uzh.ch (R.M.E.); manuela.schnyder@uzh.ch (M.S.); 6 Department of Parasitology and Zoology, University of Veterinary Medicine, 1078 Budapest, Hungary; farkas.robert@univet.hu; 7 Department of Pathobiology, Institute of Parasitology, University of Veterinary Medicine Vienna, Veterinärplatz 1, 1210 Vienna, Austria; hans-peter.fuehrer@vetmeduni.ac.at; 8 Department of Zoology, Musée National d’Historire Naturelle, 25, Rue Münster, 2160 Luxembourg, Luxembourg; mike-heddergott@web.de; 9 Infectious Disease Prepardness, Statens Serum Institut, Artillerivej 5, DK-2300 Copenhagen, Denmark; pijo@ssi.dk; 10 Clinical Unit of Internal Medicine Small Animals, Department/Universitätsklinik für Kleintiere und Pferde, University of Veterinary Medicine Vienna, Veterinärplatz 1, 1210 Wien, Austria; michael.leschnik@vetmeduni.ac.at; 11 Small Animal Clinic of Estonian University of Life Sciences, Kreutzwaldi 62, 51014 Tartu, Estonia; valentina.oborina@emu.ee; 12 Faculty of Natural Sciences, Vytautas Magnus University, K. Donelaičio str. 58, LT-44248 Kaunas, Lithuania; algimantas.paulauskas@vdu.lt (A.P.); jana.radzijevskaja@vdu.lt (J.R.); 13 Latvian Biomedical Research and Study Centre, LV-1067 Riga, Latvia; renate_r@biomed.lu.lv; 14 Centre for Infection Medicine, Institute for Parasitology, University of Veterinary Medicine Hannover, 30559 Hannover, Germany; andrea.springer@tiho-hannover.de (A.S.); christina.strube@tiho-hannover.de (C.S.); 15 Institute of Biochemistry and Biophysics, Polish Academy of Sciences, 5A Pawińskiego Str, 02-106 Warsaw, Poland; 16 Institute for Specific Prophylaxis and Tropical Medicine, Medical University of Vienna, 1090 Vienna, Austria; julia.walochnik@muv.ac.at

**Keywords:** *Babesia*, emerging, One Health, tick, vector

## Abstract

There is now considerable evidence that in Europe, babesiosis is an emerging infectious disease, with some of the causative species spreading as a consequence of the increasing range of their tick vector hosts. In this review, we summarize both the historic records and recent findings on the occurrence and incidence of babesiosis in 20 European countries located in southeastern Europe (Bosnia and Herzegovina, Croatia, and Serbia), central Europe (Austria, the Czech Republic, Germany, Hungary, Luxembourg, Poland, Slovakia, Slovenia, and Switzerland), and northern and northeastern Europe (Lithuania, Latvia, Estonia, Iceland, Denmark, Finland, Sweden, and Norway), identified in humans and selected species of domesticated animals (cats, dogs, horses, and cattle). Recorded cases of human babesiosis are still rare, but their number is expected to rise in the coming years. This is because of the widespread and longer seasonal activity of *Ixodes ricinus* as a result of climate change and because of the more extensive use of better molecular diagnostic methods. Bovine babesiosis has a re-emerging potential because of the likely loss of herd immunity, while canine babesiosis is rapidly expanding in central and northeastern Europe, its occurrence correlating with the rapid, successful expansion of the ornate dog tick (*Dermacentor reticulatus*) populations in Europe. Taken together, our analysis of the available reports shows clear evidence of an increasing annual incidence of babesiosis across Europe in both humans and animals that is changing in line with similar increases in the incidence of other tick-borne diseases. This situation is of major concern, and we recommend more extensive and frequent, standardized monitoring using a “One Health” approach.

## 1. Introduction

Babesiosis, also known as piroplasmosis, is a multisystem disease caused by the protozoan parasites of the genus *Babesia* [1,2,3,4]. Babesiosis is an important emerging infectious disease of global significance, affecting both humans and domesticated animals [5,6]. The main route of transmission of babesiae to mammalian hosts is through a tick bite, although other routes of transmission (e.g., vertical transmission, transmission through blood transfusion or organ transplantation) have been documented among both humans and animals, including also in wildlife reservoir hosts [7]. Hard ticks (Ixodidae) act as vectors of babesiae, with evidence of specificity in *Babesia*-tick vector interactions [8]. The course of babesiosis can differ considerably, from entirely subclinical infections, through mild non-specific manifestations, to fatal, multisystem disease. Additionally, there is growing evidence that the co-infection of *Babesia* and other tick-borne diseases may contribute to a more severe course of disease in patients [5]. Notably, co-infection of *Babesia microti* and *Borrelia burgdorferi* s.l. has been documented in the United States of America (USA) and Europe, with Lyme disease patients experiencing more symptoms and for a longer duration if coinfected with *B. microti* [9,10].

From a historical perspective, babesiosis has been well-described as a dangerous, potentially lethal, tick-borne disease of dogs [11,12] and cattle [13,14], the latter contributing to marked financial losses in the cattle industry. In current times, with tick control and efficient treatments for babesiosis being readily available, the disease appears to have lesser importance than in earlier periods. However, the number of human cases has been growing recently, especially in the US, with about 2000 new cases being reported annually, and in Canada and China as well [5,15,16].

The situation in Europe appears to be less acute and the disease is less recognized in the medical profession, although there are some recent reports of human cases, in particular from France, Germany, and the United Kingdom [3]. Moreover, the number of cases attributed to other important tick-borne diseases (e.g., borreliosis, rickettsiosis, tick-borne encephalitis) has been growing also and the One Health approach has been applied in investigating all of these diseases (e.g., [17,18,19,20]). Taken together, current observations suggest that there is increasing exposure of humans and animals to ticks in Europe (as reviewed for *Ixodes ricinus* in [21]).

The ornate dog tick, *Dermacentor reticulatus*, is one of the fastest-spreading tick species in central and northeastern Europe, with increasing significance as a vector of tick-borne pathogens for domesticated animals [22,23,24,25]. *Dermacentor reticulatus* is the main, if not the only, vector of *Babesia canis* and its spread is accompanied by the expansion of canine babesiosis and the appearance of new foci of the disease [8,26]. Additionally, this tick species may act as a vector of *B. caballi* and *Theileria equi* [25,27,28]. Despite focusing on *Babesia* spp. in this review, we have also included *Theileria equi*, which was originally referred to as *B. equi*, and is one of the two common piroplasm species of horses. In addition, the two piroplasms are closely related and some molecular diagnostic assays fail to distinguish between isolates of *T. equi* and *Babesia* spp. While other *Theileria* species are known to exist in wild animals, such as deer and boars, these do not pose a threat to human populations or our domestic animals in the region of Europe that is covered in our review [29]. Therefore, in the current review, we summarize recent findings on the occurrence and incidence of babesiosis in southeastern, central, and northeastern Europe (Bosnia and Herzegovina, Croatia, Serbia, Austria, Luxembourg, Switzerland, Germany, Hungary, the Czech Republic, Slovakia, Slovenia, Poland, Lithuania, Latvia, Estonia, Iceland, Denmark, Finland, Sweden, and Norway), in humans and selected species of domesticated animals (cats, dogs, horses, and cattle), and *T. equi* in horses.

## 2. South-Eastern Europe

### 2.1. Bosnia and Herzegovina (BiH)

#### 2.1.1. Babesiosis in Humans

There are no published reports to date of human babesiosis in BiH.

#### 2.1.2. Babesiosis in Animals

In June 2017 and July 2018, two cases of bovine babesiosis occurring in two small farms in central BiH were attributed to *Babesia divergens,* after confirmation by molecular methods (Polymerase chain reaction [PCR] and sequencing) [30]. In a recent molecular screening of samples from 142 horses from the Central Balkan region, including Serbia, Montenegro, and BiH, the overall prevalence was reported to be 22.5% and 2.1% for *T. equi* and *B. caballi*, respectively [31]. This study indicated for the first time that *T. equi* is actually present in BiH.

*Dermacentor reticulatus* is a tick species that is currently expanding its range in BiH. In a recent (2017–2019) monitoring study, this tick species was found in regions of the country where it had not been recorded earlier and was identified in new host species for the country [32]. Between 2014 and 2016, the DNA of *B. canis* was identified by PCR and sequencing in 85% of eighty symptomatic dogs that had never left the country [33]. Accordingly, the parasite is considered autochthonous and canine babesiosis is treated as an expanding disease in BiH. Additionally, the DNA of *B. canis* has been recorded in one red fox (*Vulpes vulpes*) (prevalence 0.8%). *Babesia vulpes* (formerly known as *B. microti*-like) (31.9%) and *Hepatozoon canis* (38.6%) have also been detected in red foxes (*n* = 119) in BiH, identified in animals hunted between October 2013 and April 2014 [34].

There are no published records of *Babesia* in domestic cats; however, *Babesia* sp. badger-type A was identified in one of 18 examined European wildcats (*Felis silvestris*) in BiH [35].

### 2.2. Croatia

#### 2.2.1. Babesiosis in Humans

The first reported case of human babesiosis in Europe, and indeed in the world, occurred in 1956 in former Yugoslavia, now Croatia. The patient was a 33-year-old tailor and part-time farmer who had been splenectomized following a traffic accident 11 years earlier [36]. Based on morphological characteristics, and the fact that the patient was a farmer, the authors concluded that the infection was caused by *Babesia bovis*. The patient presented with fever and severe hemoglobinuria eight days after first feeling unwell and died two days later. Because *B. bovis* is not known to be zoonotic, and the photomicrographs in the published case study show divergent piroplasms that are characteristic of *B. divergens*, this is currently regarded as the first human case of *B. divergens*. To date, this case remains the only confirmed case of babesiosis in Croatia, although, in 2003, a low titer of antibodies to *B. microti* was detected in a single serum from 102 patients with a history of tick bites [37].

#### 2.2.2. Babesiosis in Animals

The first records of piroplasmosis in Croatia date back to 1912, when studies on babesiosis in sheep were conducted in Dalmatia. Further studies performed by the same author in 1921 confirmed piroplasmosis in cattle, horses, and dogs from the same Croatian littoral region [38].

The results of the first molecular study were published in 2002 and in these, *B. canis* infection was confirmed in eight dogs from the Zagreb region of Croatia showing the clinical signs of babesiosis, including apathy, fever, and anemia [39]. In the second molecular study, which included 81 microscopically positive dogs with babesiosis, six piroplasm species were detected. *Babesia canis* was the dominant species, identified in 78 of the symptomatic dogs (96%), followed by single infections with *B. vogeli, B. caballi,* and *T. equi* [40]. In a group of randomly selected, apparently healthy dogs in the same study, the prevalence was 3.4% (29 of 848). Besides the confirmation of *B. canis* in 20 dogs (69%), *B. gibsoni* was detected for the first time in six dogs (21%), *B. vogeli* in two dogs (7%), and *B. vulpes* in one dog (3%). *Babesia canis* was the only species detected in dogs with lethargy, anorexia, fever, dark urine, and thrombocytopenia [41]. More recently, *B. gibsoni* has been confirmed in three dogs and *B. vulpes* in one dog showing anemia and thrombocytopenia [42]. Antibodies to *B. canis* were detected in 20% (95% CI 16.3–24.1%) of 435 randomly selected, apparently healthy dogs from 13 different locations in Croatia using indirect immunofluorescence [43].

In retrospective post-mortem studies on archived, formalin-fixed, paraffin-embedded tissue blocks (FFPEB) from dogs that had died due to a hemolytic crisis, *B. canis* was confirmed in all cases except one, *Theileria capreoli* being identified in the heart tissue of the latter case [44]. Septic shock was the cause of death in the *T. capreoli*-infected dog, based on gross and histological criteria. *Babesia canis* was confirmed by sequencing from archived Romanowsky stained cytological slides, on which positivity for canine piroplasmosis had been previously identified after microscopic examination [45]. The slides consisted of five clinical blood smears and nine different organ imprints; these were shown to be suitable for the molecular typing of the archival samples to enable species confirmation. Moreover, *B. canis* was amplified from the different tissues of 15 dogs that had shown gross findings consistent with hemolytic disease, despite clearance of the diagnostic life stages soon after treatment [45]. Among wildlife, *B. canis* has been found in grey wolves [46], *B. vulpes* in red foxes [47], and *T. capreoli* (previously *Theileria* sp. 3182/05) in grey wolves and foxes [46,47].

In 2015, Gotic [48] identified the DNA of *B. caballi* and *T. equi* in 3.6% (13/362) and 13.2% (48/362) of 362 horses, respectively. Two genotypes were detected, *T. equi* genotype E in 10.8% (39/362) of the horses and *T. equi* genotype A in 2.5% (9/362) of the horses. *Babesia caballi* and *T. equi* genotype A were found in the continental part of the country, while *T. equi* genotype E was found exclusively in coastal areas [48].

In 2020, three cows that had died, with icterus, splenomegaly, and dark urine, were found to be co-infected with *A. bovis* and *T. orientalis* [49]. In 2001, *Theileria ovis* and *Theileria* sp. OT3 were identified in both healthy and sick sheep in southern Croatia [50]. A third ovine species, *Babesia ovis,* was responsible for several cases of babesiosis in the Dubrovnik region in 2018 (unpublished data). In a single study on wild ungulates, sequence analysis of piroplasms from the spleens of red deer, fallow deer, and roe deer revealed the presence of *B. crassa, B. divergens/capreoli, B. venatorum* (previously *Babesia* sp. EU1), *T. ovis* and *T. capreoli* (*Theileria* sp. 3185/02) [51].

The DNA of *Babesia* sp. was detected in 7.1% of *I. ricinus* ticks from Zagreb, while in contrast, *B. canis* was found in 77% of *D. reticulatus* ticks from the same location [52]. In southern Croatia, the DNA of *T. ovis* was detected in the *Rhipicephalus turanicus*, while the DNA of *B. ovis* and *T. ovis* was detected in the tick, *R. bursa* [53].

### 2.3. Serbia

#### 2.3.1. Babesiosis in Humans

Both the microscopic and molecular presence of *Babesia* in human blood samples still remains unconfirmed among Serbian citizens, despite a broad “One Health” approach study on tick-borne diseases in Serbia [54].

#### 2.3.2. Babesiosis in Animals

The first records of seasonal hemoglobinuria associated with tick infestations in grazing cattle, sheep, goats, and horses on Serbian territory were recorded in the 19th century. Although piroplasms were not established as the etiological cause of disease in this case, the authors recommended prevention measures aimed at ticks in order to decrease exposure to infection. The first microscopic evidence of piroplasms in dogs from Serbia was published in 1953 [38]. Awareness of the importance of canine piroplasmosis, based on clinical and microscopic diagnoses by veterinarians from Belgrade and Novi Sad working in the domain of “small animal practice”, was raised during the early 1980s [55]. Available data indicated that the Belgrade district was not a highly endemic region for canine babesiosis at that time. Sporadic cases of babesiosis were recorded in hunting and companion dogs that had returned from vacations. In their study of 1997–2001, Pavlović et al. [56] examined a total of 3945 dogs with febrile disease accompanied by anemia, hemoglobinuria, general pallor, and icterus or tick infestation. In this study, *B. canis* was found in 74% of blood smears, confirming its endemic status in the region of the Serbian capital. The prevalence of infection was observed to increase between March and April and then to decrease by July. A second peak occurred in September and additional cases continued to appear until December [56]. In a similar study conducted in 2015–2017 on 1085 dogs with overt signs of babesiosis, prevalence by microscopy was 35%, half that recorded in the earlier study [57]. Morphological appearance allowed for the identification of *B. canis* in 95% of infected animals. Another study in 2013–2016 confirmed the endemic status and seasonal appearance of canine babesiosis due to *B. canis* in Belgrade [58].

In 2012–2013, in the region of Vojvodina (northern Serbia), *Babesia* spp. were diagnosed by microscopy in 12.2% of 41 dogs suspected of having babesiosis [59].

The first molecular study on *Babesia* species in Serbia was published in 2015; this was based on 60 dogs with clinical signs of babesiosis [60]. For the first time, *B. canis* and *B. gibsoni* were identified in Serbian dogs by PCR-RFLP and PCR and sequencing. In a more recent study encompassing 111 dogs, including 46 from shelters, 31 free-roaming, and 34 hunting dogs, *B. canis* and *B. gibsoni* were detected in 16.2% of the dogs through species-specific real-time PCR. None of the dogs tested positive for *B. vogeli* [61]. Furthermore, *B. canis* was confirmed by PCR (tick/vector comprehensive real PCR panel—canine) to be the only species found in 29 dogs microscopically diagnosed with babesiosis in Belgrade [62].

During 2012 to 2014, an overall *Babesia* prevalence of 21.5% was found in 158 healthy, asymptomatic, outdoor dogs originating from Pančevo and Ðurđevo (northern Serbia) and Niš and Prokuplje (southern Serbia). Five species of piroplasms were confirmed by sequencing: *B. vulpes* (10.1%), *B. gibsoni* (5.7%), *B. vogeli* (1.9%), *B. caballi* (1.9%), and *B. microti* (1.9%). Dogs from Prokuplje were more frequently infected (59.1%) than dogs from Panćevo (11.9%) or Niš (4.5%). No infected dogs were found in Ðurđevo [63].

So far, only a single study has been conducted on equine piroplasmosis in Serbia [31]. In 2014, 94 apparently healthy horses from four locations were examined with multiplex PCR and sequencing. The overall prevalence was 28.7% and the predominant species was *T. equi* (27.7%), with a single sample testing positive for *B. caballi* (1.1%). The prevalence of EP varied within a range of 0–7.7% between collection sites in three NW counties and 92.3% in the single SE county included in this survey. The prevalence was associated with the exposure of farming horses to ticks when grazing throughout most of the year [31]. The similar high prevalence of *T. equi* (50%) was found in 70 blood samples from apparently healthy donkeys [64]. Hematological alterations were detected in 54% of the tested donkeys, while the DNA of *T. equi* was detected in 92% of donkeys with hematological abnormalities. Interestingly, *B. caballi* was not detected in these donkeys.

PCR testing to investigate bovine piroplasmosis was conducted by Vasic and co-workers [65], who detected *Theileria* spp. in 5 out of 135 bovine blood samples from northern and central Serbia. Sequences were 100% identical with GenBank entries from Italy (*Theileria sergenti*), China (*Theileria* spp.), and Korea (*Theileria buffeli* isolate HS252). The blood samples had been collected during May and June of 2013 from six geographically different locations in the northern to southern regions of Serbia. All the infected animals were found to have originated from Banatski Karlovac, northeastern Serbia, and were free of any clinical signs that could be related to theileriosis.

*Babesia canis* was confirmed by PCR and sequencing in 4.2% (9/216) of golden jackals [66]. In red foxes, two *Babesia* species were identified by molecular methods: *B. vulpes* (28.7%) and *B. canis* in a single fox (0.8%) [67]. Co-infection with *B. vulpes* and *H. canis* was present in 20.2% of foxes.

In several studies, both zoonotic (*B. microti, B. venatorum*) and non-zoonotic (*B. canis*) *Babesia* species have been detected in ticks collected from vegetation and animals in Serbia [54,68,69].

## 3. Central Europe

### 3.1. Austria

#### 3.1.1. Babesiosis in Humans

To date, three cases of human babesiosis have been described in Austria. Two of these cases were attributed to *B. venatorum*, including one in a 56-year-old splenectomized huntsman who remembered a tick-bite two weeks before the onset of symptoms [70], and one in a splenectomized 68-year-old male patient with acute renal failure, whose serum revealed an anti-*Babesia* antibody titer of 1:1024 in an immunofluorescence assay [71]. The third case occurred in a non-splenectomized 63-year-old male patient who had spent four weeks in Massachusetts, USA, occupied in mainly outdoor activities shortly before the onset of symptoms. In this case, the causative agent was identified as *B. microti* by PCR, and sequencing [72].

In a recent unpublished study, 1253 hunters and 414 other individuals, each of the latter with a history of tick bites, were screened for antibodies against *Babesia* spp. with an in-house immunofluorescent antibody test (IFAT) using Fluoline H conjugates (Biomerieux, Vienna, Austria). Of the hunters and individuals with a history of tick bites, 101 (8.1%) and 35 (8.4%), respectively, tested positive for *Babesia* spp. The age range of positive humans was 17–73 years (mean 54.02) and titers ranged from 1:16 to 1:256. Additional blood samples were obtained from all serologically positive individuals who were available for follow-up and were investigated by a commercial PCR. Altogether, seven individuals were found to be positive for *Babesia* spp. by PCR, and in one of these individuals, the intra-erythrocytic stages of *Babesia* sp. were also detected microscopically. Five of the PCR-positive individuals were symptomatic, while three were co-infected with *Borrelia* spp. The sequencing of PCR products revealed *B. venatorum* and *B. microti*. One sequence could not be identified because of poor sequence data.

#### 3.1.2. Babesiosis in Animals

Several *Babesia* species, including *B. canis*, *B. capreoli*, *B. divergens*, *B. microti*, *B. venatorum*, and *B. vulpes* have been detected in Austrian pets, livestock, and wildlife animals, but also in ticks [73,74,75,76,77,78,79,80]. In a large study of *Babesia* in ticks, the most prevalent *Babesia* species were from the *B. divergens*/*B. capreoli* cluster [73].

Autochthonous babesiosis has become a common disease in dogs in Austria but is absent in cats. The presence of *B. canis* is thought to be focused in the eastern Austrian regions where *D. reticulatus* is endemic [81,82]. In some of these endemic areas, up to 25% of *D. reticulatus* ticks are infected with *B. canis* [76]. Canine babesiosis became endemic within the last 30 years, possibly introduced by hunting dogs that frequently crossed the border from western Hungary. Nowadays, autochthonous cases are reported especially from areas of eastern Austria at low altitude levels (below 800 m), thus representing a suitable habitat for these ticks [83]. In a recent study, the DNA of *B. canis* was detected in six out of 94 (6.4%) clinically healthy military dogs kept in kennels in Burgenland (Eastern Austria) [84].

Another study reported several cases from alpine valleys in Tyrol and Carinthia in western and southern Austria [85]. Strobl et al. [86] determined the proportion of autochthonous canine cases with clinical signs in Austria to be 59.6%. A study from Eastern Austria calculated an annual risk for infection of 6.9% for dogs, leading to clinical signs in 50% of the infected animals [87]. A fatality rate of 10%, independent of the therapeutic measures applied, has been recorded in canine babesiosis within the last decades in Eastern Austria. Infection has also been documented in a red fox (*V. vulpes*) (1/351 blood samples; 0.3%) but it was concluded that red foxes are unlikely to have a significant impact as a reservoir or as spreader hosts [34,82].

*Babesia gibsoni* was found in 2015 in a dog with a history of tick infestation from the “Mauererwald” forest, close to Vienna (Prof. Walter G. Url, personal communication), and also more recently [88]. The last case attracted some interest from researchers because it was a co-infection of *B. canis* with *B. gibsoni* in a dog imported from Serbia [88]. However, it is still unclear whether *B. gibsoni* is currently endemic in Austria.

In Austria, foxes are acknowledged as the main hosts of *B. vulpes* (formerly known as *B. microti*-like or “*Babesia* sp. from Spanish dog”), a parasite known to rarely infect dogs [89]. Various studies have revealed a high prevalence of *B. vulpes* in foxes in western and eastern Austria [82,90].

Cattle are mainly affected by *B. divergens*, a species that is widespread in the Austrian alpine regions [74]. Between 1998 and 2016, a total of 1257 fatal cases of babesiosis were reported in the federal province of Styria (southeastern Austria), with high-risk clusters in the central, northern, and western regions of Styria [91]. *Babesia divergens* has also been documented in red deer populations in Styria (1/37; 2.9%) and Tyrol (10/196; 5.1%) [79,92].

Autochthonous cases of *B. caballi* have not as yet been recorded in Austria. However, an autochthonous case of *T. equi* was reported recently in its easternmost province (Burgenland) in a horse and in *D. reticulatus* [27].

### 3.2. Czech Republic

#### 3.2.1. Babesiosis in Humans

To date, only two cases have been reported from the Czech Republic (CR). The first case of human babesiosis was recognized in 2000 [93], being also the first case of a symptomatic *B. microti* infection imported from the USA to Europe. *Babesia microti* infection was diagnosed on the basis of a positive blood smear and antibody detection by IFAT [93].

Recently, babesiosis was diagnosed and successfully treated in a patient initially diagnosed with Reiter’s syndrome because of symptoms corresponding to the classic triad of arthritis, conjunctivitis, and non-specific urethritis [94]. This was also the first suspected case of post-transfusion babesiosis, as the patient, a 36-year-old male who had experienced a motorcycle accident with consequent severe polytrauma, received repeated blood transfusions. Six months following the transfusions, the patient suffered from non-specific symptoms, including dysuria and periurethral itch, mild edema and itching of the eyelids, and joint pains. The abdominal ultrasonographic examination revealed mild splenomegaly and slight hepatomegaly, with normal echogenicity and without focal changes in the parenchyma. The diagnosis of babesiosis due to *B. microti* was made by an immunoassay, the lymphocyte transformation test (LTT, ELISPOT) [94].

#### 3.2.2. Babesiosis in Animals

Despite the widespread occurrence of *B. divergens* in red deer and, to a lesser extent, in sika deer, no bovine babesiosis cases have been reported from the CR for many decades now [95]. Moreover, no *Babesia* DNA was detected among the 100 blood samples collected in 2014–2015 from cattle [95].

In a recent study performed in 2014–2018, blood and serum samples were collected from 711 healthy horses [96]. Antibodies to *T. equi* were detected by cELISA in eight (1.1%) horses and antibodies to *B. caballi* in three (0.4%) horses that were also positive for *T. equi*. Seropositivity to *T. equi* and *B. caballi* was confirmed by IFAT in five and three of the horses, respectively, that had shown positivity via cELISA. Samples that were seropositive for *T. equi* (*n* = 8) and *B. caballi* (*n* = 3) were then tested by PCR; the DNA of *T. equi* was identified in five horses, but no *B. caballi* DNA was detected in any of the serologically positive horses.

Canine babesiosis is considered to be an emerging disease in the CR, following the ongoing expansion of the range of *D. reticulatus* ticks in Europe [97]. Until recently, babesiosis caused by *B. canis* was reported frequently in the CR but only as an imported disease [98,99]. Then, in a group of 41 non-traveling dogs from the South Moravian region, 12% of the dogs tested seropositive, although the DNA of *B. canis* was not detected either in the examined dogs or in 340 questing *D. reticulatus* ticks collected in the region [100]. South Moravia, in the southeastern region of the CR, has been known for over 50 years to be an endemic area for *D. reticulatus* [101,102], and ticks of this species were found in 2009–2010 in numerous localities in South Moravia [103]. However, the DNA of *B. canis* was identified in shelter dogs from this region of the country only in 2017, and the first clinical autochthonous case of *B. canis* was diagnosed in a non-traveling dog from this area one year later [104,105]. The most recent study of the distribution of *D. reticulatus* in the CR, based on a citizen science campaign of 2018–2021, revealed that *D. reticulatus* is actually present in all regions of the CR. This work suggested a real risk of emergence of canine babesiosis in at least two regions with well-established tick populations, in the southeastern and northwestern CR [97].

In 2017, *B. gibsoni* was confirmed by PCR-sequencing in an American pit bull terrier with clinical signs of acute babesiosis [104]. The dog originated from Slovakia, where *B. gibsoni* was reported for the first time in two pit bull terriers in 2013 [106]. No cases of babesiosis in cats have been reported to date from the CR [12].

### 3.3. Germany

#### 3.3.1. Babesiosis in Humans

Among the zoonotic *Babesia* spp., *B. divergens*, *B. microti*, *B. venatorum* and *B. motasi* have been reported in Germany. *Babesia microti* was first identified in European field voles in Germany in the 1970s, but autochthonous human infections were not yet known at the time [107]. The first confirmed autochthonous clinical case due to *B. microti* in Europe was reported in a German patient with myeloid leukemia in 2007, and the source of infection was attributed to a blood transfusion [108]. In one of the donors from whom the patient had received blood products, a borderline *B. microti* IgG titer was demonstrated but active infection could not be confirmed. Neither the patient nor the blood donor had traveled to North America or Asia. The obtained 284-bp 18S rDNA sequence from the leukemia patient showed 100% identity with the Gray strain [108]. A second clinical case involving *B. microti* in Germany has been published, but the infection was acquired in North America [109]. One further autochthonous babesiosis case was reported in 2007 in a splenectomized and immunocompromised patient, and the causative agent was identified as *B. venatorum* (known as *Babesia* sp. EU1 at the time) [110]. Human *B. divergens* infections have not as yet been reported in Germany [3].

The first serological study on *Babesia* exposure was conducted by Krampitz et al. [111], who detected a *B. microti* seroprevalence of 0.25% among 798 healthy forestry workers from the federal state of Bavaria in southern Germany. In the same study, four sera (0.5%) were positive for *B. divergens* antibodies by ELISA but not by IFAT. Further studies in the following years indicated seroprevalence values of 1.7–13.9% for *B. microti* [112,113,114] and 0.8–4.9% for *B. divergens* [112]. In general, higher values were found in risk groups (forestry workers, Lyme borreliosis patients, and humans exposed to ticks) rather than in non-risk groups (Table 1). Exposure to *B. venatorum* or *B. motasi* has not been studied. *Babesia motasi* has been implicated in human babesiosis cases in Asia [5]. This ovine piroplasm was detected in herds of sheep in northwestern and central parts of Germany in the 1950s [115]. However, to our knowledge, there are no recent reports of *B. motasi* in Germany, neither in sheep nor in humans.

In summary, only three human babesiosis cases have been reported in Germany, two being autochthonous and one imported, and all three patients were splenectomized and/or immunocompromised. In contrast, high seroprevalence values of anti-*Babesia* antibodies have been found, particularly in high-risk groups such as forestry workers. This may indicate that *Babesia* strains circulating in Germany show low pathogenicity, or that the disease is underreported.

#### 3.3.2. Babesiosis in Animals

Two causative agents of bovine babesiosis occur in Germany, *B. divergens* and *B. major*. However, the latter is only present on certain islands where the tick vector *Haemaphysalis punctata* occurs [116]. Reports of babesiosis in German cattle are rare, and most studies date from the 1980s. At the time, seroepidemiological investigations were conducted in the federal state of Bavaria in southern Germany, resulting in *B. divergens* seroprevalence rates of 13.0–21.0%, with considerable differences between districts [117,118] (Table 1). Furthermore, serological investigations have been conducted on farms with the suspicion or a history of bovine babesiosis that are located in the federal state of Lower Saxony in northern Germany, where seroprevalences of 4.0–43.0% were recorded [119,120] (Table 1).

**Table 1:** Summary of studies reporting the (sero-)prevalence of *Babesia* spp. in Germany.

Reference	Host Species/Group (No. of Individuals Examined)	*Babesia* Species (No. of Cases)	*Babesia* Species (No. of Seropositive)	Prevalence/Seroprevalence
Krampitz et al. 1986 [111]	Humans, healthy forestry workers (798)	n.a.	*B. microti* (2)	0.25%
		n.a.	*B. divergens* (4)	0.5%
Hunfeld et al. 1998 [114]	Humans, Lyme borreliosis patients (76)	n.a.	*B. microti* (9)	11.8%
	Humans, seropositive but asymptomatic Lyme patients (44)	n.a.	*B. microti* (4)	9.1%
	Humans, syphilis patients (50)	n.a.	*B. microti* (2)	4.0%
	Humans, healthy blood donors (100)	n.a.	*B. microti* (8)	8.0%
Hunfeld et al. 2002 [112]	Humans exposed to ticks (225)	n.a.	*B. microti* (21), *B. divergens* (11)	*B. microti:* 9.3%, *B. divergens:* 4.9%
	Humans with various infectious diseases (122)	n.a.	*B. microti* (2), *B. divergens* (5)	*B. microti:* 1.6%, *B. divergens:* 4.1%
	Humans, healthy blood donors (120)	n.a.	*B. microti* (2), *B. divergens* (1)	*B. microti:* 1.7%, *B. divergens:* 0.8%
Scheller 2004 [113]	Humans, forestry workers with fever (490)	n.a.	*B. microti* (68)	13.9%
Weiland et al. 1980 [117]	Cattle (1220)	n.a.	*B. divergens* (256)	21.0%
Ullmann et al. 1984 [118]	Cattle (1616)	n.a.	*B. divergens* (211)	13.1%
Ganse-Dumrath 1986 [119]	Cattle from farms with history of babesiosis (251)	*B. divergens* (29)	*B. divergens* (108)	43.0%
Niepold 1990 [120]	Cattle, *Borrelia*-positive animals (212)	n.a.	*B. divergens* (0)	0.0%
	Cattle, farms with suspected babesiosis (354)	n.a.	*B. divergens* (0)	0.0%
	Cattle, farms with history of babesiosis (200)	n.a.	*B. divergens* (8)	4.0%
Huwer et al. 1994 [121]	Cattle, farms with babesiosis history (187)	*B. divergens* (14)	*B. divergens* (88)	47.1%
Lengauer et al. 2006 [122]	Cattle (287)	n.a.	*B. divergens* (1)	0.3%
Springer et al. 2020 [123]	Cattle, one farm with history of babesiosis (95)	*B. divergens* (30)	*B. divergens* (36)	37.9%
Pikalo et al. 2016 [124]	Horses (314)	n.a.	*T*. *equi* (19), *B*. *caballi* (1)	*T. equi*: 6.1%, *B. caballi*: 0.3%
Boch 1985 [125]	Horses (321)	n.a.	*T. equi* (18), *B. caballi* (4)	*T. equi*: 5.6%, *B. caballi*: 1.2%
	Dogs with suspected babesiosis (116)	n.a.	*B. canis* complex * (46)	39.7%
Hirsch and Pantchev 2008 [126]	Dogs, imported or traveling (5142)	*Babesia* spp. (n.a.)	n.a.	2.1–2.7%
Menn et al. 2010 [127]	Dogs, imported or traveling (4681)	n.a.	*B. canis* complex * (1138)	24,3%
Hamel et al. 2011 [128]	Dogs, traveling (648)	n.a.	*B. canis* complex * (32)	4.9%
	Dogs, traveling (508)	*B. canis* complex * (19)	n.a.	3.7%
Röhrig et al. 2011 [129]	Dogs, imported (2819)	n.a.	*B. canis* complex * (251)	8.9%
	Dogs, imported (2288)	*B. canis* complex * (5)	n.a.	0.5%
Pantchev [130]	Dogs, imported or traveling (4579)	n.a.	*B. canis* complex * (319)	7,0%
	Dogs with suspected babesiosis (937)	n.a.	*B. canis* complex * (119)	12.7%
Liesner et al. 2016 [131]	Dogs (1023)	*B. canis* (1)	n.a.	0.1%
Vrhovec et al. 2017 [132]	Dogs (9966)	*B. canis* complex * (170)	n.a.	1.7%
	Dogs (15,555)	*Babesia* spp. (502)	n.a.	3.3%
	Dogs (2653)	n.a.	*Babesia* spp.	11.5%
Schäfer et al. 2019 [133]	Dogs, imported (98)	*Babesia* spp. (3)	n.a.	3.1%
	Dogs, imported (214)	n.a.	*B. canis*/*B. vogeli* (22)	10.3%
Schäfer et al. 2019 [134]	Dogs, traveling (127)	*Babesia* spp. (3)	n.a.	2,4%
	Dogs, traveling (160)	n.a.	*B. canis*/*B. vogeli* (8)	0.5%
Schäfer et al. 2021 [135]	Dogs (20,914)	*Babesia* spp. (659)	n.a.	3.2%
	Dogs, never been abroad (692)	*Babesia* spp. (54)	n.a.	7.8%

n.a.—not applicable. * Note that the *B. canis* complex was previously classified as a complex of *B. canis*, *B. vogeli* and *B. rossi* as subspecies.

As *B. divergens* antibody titers show strong seasonal variation [121,136], the timing of sample collection with respect to the grazing season may explain the large variation in these results. Furthermore, *B. divergens* seems to occur in a focal pattern. This is exemplified by the study of Huwer, Schwarzmaier, Hamel and Will [121], who located three valleys in the federal state of Baden-Wuerttemberg (near Freiburg i. Br.), where bovine babesiosis occurred. Since 2000, reports of bovine babesiosis in Germany are rare. A decline in bovine babesiosis seroprevalence or incidence has been reported in other European countries [137,138], but comparable data from Germany are lacking. However, a recent report illustrates that the introduction of infected ticks or animals may lead to *B. divergens* (re-)emergence, with considerable economic losses for farmers. In this case, 25 animals died of bovine babesiosis on an affected farm between 2018 and 2019 [123]. Seroepidemiological investigations revealed that only one herd, which had grazed on a particular pasture, was affected during the first year of the outbreak, whereas events during the second year suggested that infected ticks had spread further afield on the farm [123].

Currently, equine piroplasmosis (EP) is considered to be non-endemic in Germany, although competent vectors, such as *D. reticulatus* and D. *marginatus*, are present [22]. Therefore, the importing of infected horses or ticks from endemic countries poses a risk of becoming endemic. As no import restrictions exist with regard to EP, seropositive horses frequently enter Germany. Between 1997 and 1999, 19 of 42 *B. caballi*- and/or *T. equi*-seropositive horses that were diagnosed at the Ludwig-Maximilians-University, Munich, were imported or had a travel history, whereas information was lacking for the remaining animals [139]. A more recent study detected 6.1% of *T. equi*- and 0.3% *B. caballi*-seropositive animals among 314 German horses where the travel history was not reported [124].

Among the published clinical cases, two involved *B. caballi* and eight, *T. equi* [27,30,140,141]. Five infections were imported and four were considered to be autochthonous, while the situation was unclear in a further case. The first, presumably autochthonous, case involved *T. equi* and was reported in 2003 in a mare that had only left Germany once to visit the Netherlands, several years prior to the onset of illness [140]. The authors discussed the introduction of infected ticks, such as *Hyalomma* spp., as a possible source of infection. Indeed, *Hyalomma* spp. are increasingly found on horses in Germany during particularly warm and dry summers [142]. A further case involved a gelding that originated from France and had traveled to various EP-endemic areas earlier in life [143]. Therefore, this case may represent a reactivated *B. caballi* infection. Furthermore, two *T. equi* infections imported from France were reported in 2020 [141]. Dirks et al. [27] summarized six EP cases diagnosed in Germany between 2014 and 2019: three were presumably autochthonous, while two infections were subclinical and had probably been acquired before the importation of the horses from Russia. For the remaining animal, no travel history was provided [27]. In summary, the number of autochthonous EP cases currently seems to be increasing in Germany.

The first reported case of canine babesiosis in Germany dates back to 1909, when four dogs in a fox-terrier kennel were affected within a few weeks [144]. Further reports followed from the mid-1970s onward, and by the end of this decade, 32 cases had been diagnosed in traveling or imported dogs, 31 of which were caused by *B. canis* and one by *B. gibsoni* (summarized in [145]). In the 1980s, the number of reported *Babesia* infections in dogs increased [125,146,147], and in 1989, the first autochthonous infections and the existence of an endemic focus were postulated in the literature, after 70 dogs from the area of Offenburg, a federal state of Baden-Wurttemberg, southern Germany, were diagnosed with *B. canis* without having been abroad. Up until the early 21st century, further endemic *B. canis* foci were discovered in the southern German federal states of Baden-Wurttemberg, Bavaria, Rhineland-Palatinate, and Saarland [146,148,149,150,151,152], while only a few sporadic autochthonous *B. canis* cases were reported from dogs in the northern part of Germany [151,153,154].

At the same time, two cases of *B. gibsoni* (Asian genotype) infection were reported in American pit bull terriers that had been presented independently to the same veterinarian. Both dogs were born and lived in the county of Ravensburg, southern Germany, and had never been abroad [155]. The dogs had no known contact with each other, nor was there any history of dog-fighting or blood transfusions. The authors also considered transplacental transmission in both dogs to be unlikely and, therefore, described the cases as the first two autochthonous *B. gibsoni* infections in Germany [155]. Regarding new imported pathogens, a newly detected *B. microti*-like species, for which the name *B. vulpes* has since been proposed [156], was described in a German dog that had acquired the infection in 1994 during a stay in the Pyrenean region of Spain [150].

The first major epidemiological datasets on canine babesiosis include data from the first decade of the 21st century. These retrospective studies (listed in Table 1) included several thousand diagnostic samples from imported or traveling dogs, between 2004 and 2008, and reported 0.5–3.7% blood-smear-positive [128,129,132] and 2.1–3.3% *Babesia* spp. PCR-positive dogs [126,132]. The indirect detection of antibodies against the *B. canis* complex (previously classified as a complex of *B. canis*, *B. vogeli,* and *B. rossi* as subspecies) resulted in 4.9–24.3% of seropositive dogs [127,128,129,132]. Similar values have been found in recent studies covering the period from 2007 to 2018. *Babesia canis*/*B. vogeli* seroprevalences were 0.5–10.3% [130,133,134] in imported and traveling dogs and 12.7% in suspected cases of babesiosis [130]. Additionally, two out of 35 dogs tested positive for antibodies against *B. gibsoni* [133,134]. *Babesia* spp. DNA was found in 0.1–3.1% of the dogs [52,53,54]. Sequencing was reported from five samples only, three of which were identified as *B. canis* [131,133] and two as *B. gibsoni* [134].

Currently, *D. reticulatus* shows a continuing range expansion in Germany and has now colonized the entire national territory [22]. The authors warned that this spreading is of major importance for veterinarians and dog owners in terms of canine babesiosis outbreaks or endemic status in what were hitherto *B. canis*-free areas [22]. Shortly thereafter, an increase in autochthonous cases of canine babesiosis in the federal states of Berlin/Brandenburg, northern Germany, was reported [157]. While only five autochthonous *B. canis* infections were diagnosed in 2015–2016, there were 20 cases in 2019–2021. Similarly, 77 autochthonous canine babesiosis cases were reported between 2018 and 2020 in the Rhine-Main area of the federal state of Hesse, which was not previously known as a *B. canis*-endemic focus [158]. Increasing numbers of autochthonous *B. canis* infections are also evident from a recent study analyzing the PCR results of diagnostic dog samples from 2007 to 2020 [135]. Of the total of 20,914 samples screened, 3.2% (659 samples) were positive for *Babesia* spp., mostly *B. canis* (199/205 identified samples). This percentage is comparable to previous results [126,131,132,133,134]; however, a somewhat high percentage of 7.8% (54/692) of dogs that had not stayed abroad tested PCR-positive, and high incidence correlated with areas of high levels of activity of *D. reticulatus* in Germany [22], indicating that autochthonous *B. canis* infections occur in considerable numbers in dogs in Germany [135].

The only case of feline babesiosis so far in Germany was reported in 1997 [159]. A 10-month-old Norwegian Forest cat, presenting with fever, anemia, and icterus, was imported from Sweden 7 months prior and, ever since, had lived exclusively in its new home area. The morphology of the *Babesia* in the blood smear roughly corresponded to those described in a Siamese cat in Zimbabwe [160].

### 3.4. Hungary

#### 3.4.1. Babesiosis in Humans

To date, no reports of human babesiosis in Hungary have been published so far. The first likely autochthonous case of babesiosis due to *B. microti* was diagnosed by PCR in 2021 in a 64-year-old man from the southern part of Hungary (Farkas, personal communication).

#### 3.4.2. Babesiosis in Animals

The presence of equine piroplasmosis in Hungary, caused by *B. caballi,* was first described by Buza et al. in the 1950s [161,162]. Half a century later, a serological survey by cELISA of 371 horses, kept in 23 different locations, confirmed the stable endemic focus of this piroplasm in the same area of the country, in Hortobágy, where it was first detected [163]. These authors also reported the first serological evidence of horses naturally infected with *B. canis* in seven regions of Hungary. No information had been available about the other species until 2004, when the first autochthonous case caused by *T. equi* was identified in a horse with clinical signs of piroplasmosis [164]. A few years later, the first serological and molecular study on *T. equi* infection in horses was carried out [165]. The results indicated that the parasite was present subclinically in the western as well as in the eastern parts of Hungary. Based on these findings, the prevalence of *T. equi* was much higher than expected and the species was present in many regions of the country, unlike *B. caballi*.

According to unpublished data from the period between 1958 and 1967, there were several endemic foci of bovine babesiosis caused by *B. divergens* in northeastern Hungary [166]. At that time, the number of clinical cases was fluctuating, with intervals of 4–5 years and monophasic seasonality, peaking in June. Although Kotlán et al. [167] reported that some cases were attributed to *B. bigemina,* its main vector, *Rhipicephalus* spp., had not been identified by that time in Hungary. Therefore, these infections were most likely caused by *B. major*, transmitted by *H. punctata*. In a more recent study of 654 cattle kept outdoors, grazing in potential tick habitats in or near the endemic area, only two individuals had antibodies to *B. divergens*. The results of this first report on the prevalence of *B. divergens* infection in cattle suggest that this *Babesia* species was becoming extinct in northeastern Hungary [138]. This dramatic change could have been triggered by the massively reduced population of cattle and possibly also because fewer susceptible animals had been imported into the region. The last case of known clinical *B. divergens* infection was diagnosed in May 2008 (unpublished result). A few years later, however, the first evidence for the occurrence of *B. major* and *T. buffeli* in local cattle was reported [168]. In a herd of 88 beef cattle from northeastern Hungary, *B. major* was identified in five animals, two of which died, while four cattle harbored *T. buffeli*, of which one was anemic. In another study, *T. orientalis* was identified in 20 blood samples from 90 dairy cattle in northern Hungary, the first report of this piroplasm species in Europe [169]. This report also provided the first molecular evidence of the presence of piroplasms of sheep and goats in Hungary (*B. motasi* in central–eastern Europe, and a *B. crassa*-like strain in Europe), which were detected in the tick species *Haemaphysalis concinna* and *H. inermis*, respectively.

Based on the reported data, *B. canis* is endemic in Hungary. Autochthonous canine babesiosis in Hungary was first described more than a century ago [170]. A few decades later, Miklósi [171] wrote about piroplasms in the blood samples of three hunting dogs, identified by microscopy. Further autochthonous cases have been reported since then [172,173], based on the clinical picture of babesiosis in dogs. Microscopic evaluation of the blood smears has demonstrated the presence of the large (3–5 µm), pyriform, frequently paired parasites. Clinical and hematological findings thus indicated that *B. canis* is endemic in some parts of Western Hungary and Budapest. Csikós et al. [174] observed the well-documented seasonality of clinical babesiosis in dogs, with peaks around March–April and October–November, in an eight-year-long study of the occurrence/outbreaks of the disease. Two studies [175,176] have reported that the main vector species, *D. reticulatus*, occurs in a greater geographical range than Horváth and Papp [172] had previously described.

Until 2005, the identification of piroplasms had been based merely on the size and morphology of the intraerythrocytic parasites. The first molecular survey on natural *Babesia* infections in dogs in Hungary, using PCR and sequence analysis, was attempted subsequently to clarify the species (genotype) and to obtain information on the occurrence of *B. canis*. A piroplasm-specific PCR amplifying the partial 18S rRNA gene yielded an approximately 450 bp PCR product in 39 (88.6%) of 44 blood samples from dogs with acute babesiosis. This was the first molecular confirmation of *B. canis* in Hungary [177]. Thirteen positive samples originated from Budapest and 26 came from 21 other locations. The geographical origin of the PCR-positive samples indicated the presence of piroplasms in many districts of Budapest and in several other parts of the country, including northeastern and southeastern regions, from which no babesiosis cases had been reported previously.

Hornok et al. [178] reported first on the seroprevalence of canine babesiosis in Hungary. In total, 651 blood samples were collected from urban and rural dogs in various parts of Hungary to measure antibody levels for *B. canis* by IFAT. Thirty-seven (5.7%) sera were positive, with titers of between 1:80 and 1:10,240. Seroconverted dogs were found in 13 locations in the country, indicating that canine babesiosis was becoming more prevalent in Eastern Hungary.

The first reports on the occurrence of small *Babesia* spp. in dogs in Hungary were based on blood and splenic impression smears [179,180]. However, their species status remains uncertain, due to the lack of molecular identification, especially since *B. canis* can also exhibit small *Babesia* spp.-like morphology, depending on the sampling conditions [181]. Recently, a badger-associated *Babesia* sp. DNA was detected for the first time in dogs used for hunting, one of which showed the relevant clinical signs [182].

The most recent data on small *Babesia* spp. in dogs were reported by Tuska et al. [183]. In this study, blood samples from 79 American Staffordshire Terrier dogs, confiscated from their owners for participation in illegal dog fights, were molecularly analyzed for tick-borne pathogens. *Babesia gibsoni* was detected in 32 dogs (prevalence of 40.5%). Based on a partial fragment of the 18S rRNA gene, *B. gibsoni* from Hungary exhibited complete sequence identity with conspecific strains reported from Europe and Asia. There are only sporadic cases of *B. gibsoni* infections in the country, partly due to the absence of indigenous status of the main vector, *Rhipicephalus sanguineus* sensu stricto (s.s.). However, recently, the emergence and establishment of this tick species have been reported in the country [184]. *Babesia vulpes* has also been found in dogs in Hungary (*n* = 8; prevalence of 10.1%), where previously this piroplasm had only been reported in red foxes (*V. vulpes*) [185].

So far there have not been any published reports of autochthonous or imported babesiosis in cats from Hungary.

### 3.5. Luxembourg

#### 3.5.1. Babesiosis in Humans

There are no reports of human babesiosis from Luxembourg in the literature [186]. To the best of the authors’ knowledge, no specific studies on babesiosis in humans have been conducted to date in Luxembourg. The only study on all relevant tick-borne pathogens was performed in 2007 by PCR on ticks of the species *I. Ricinus* collected from all regions of Luxembourg [187]. According to this study, *Babesia* spp. were detected in 2.7% (*n* = 37) of *I. ricinus* ticks, with *B. venatorum* being predominant (59.5%), and *B. microti* being the second most common species (35.1%). *Babesia divergens* and *H. canis* were each detected in only one tick (2.7%).

#### 3.5.2. Babesiosis in Animals

**Figure 1:**
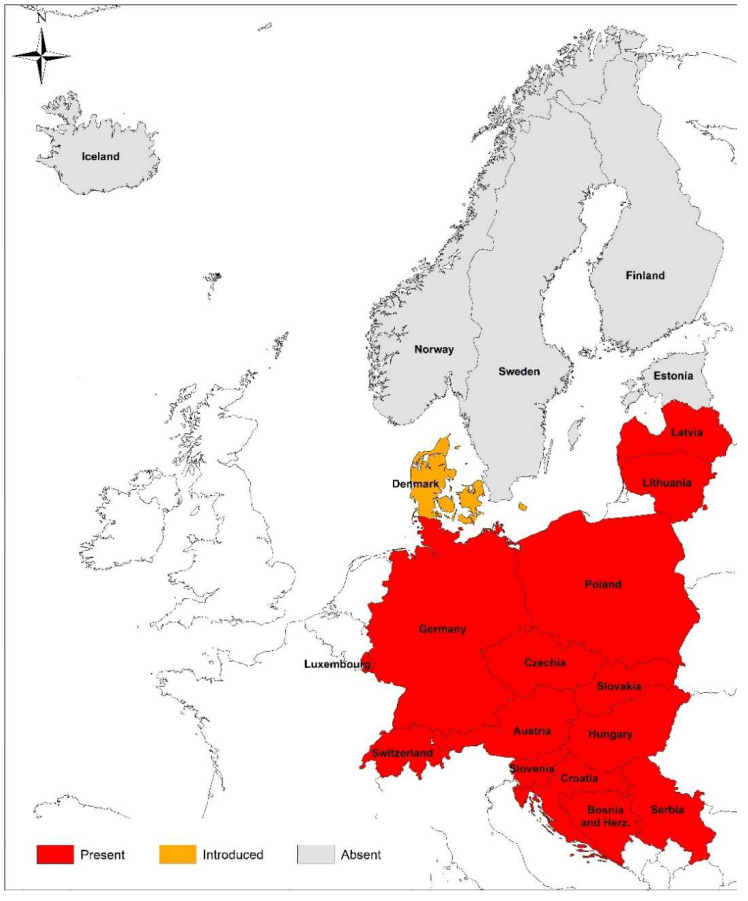
Occurrence of the ornate dog tick, *Dermacentor reticulatus,* in the reviewed countries according to https://www.ecdc.europa.eu/sites/default/files/images/Dermacentor_reticulatus_2021_09.png (accessed on 20 March 2022).

Similarly, thus far, there is no report in the literature on babesiosis in animals from Luxembourg. To date, only one unpublished case of babesiosis in a stray cat (Heddergott, personal communication) is available from Luxembourg. According to this, a road-killed 7-year-old male cat from the administrative district of Echternach in Northern Luxembourg was found to have *B. canis*-like piroplasm in a blood smear. However, PCR-sequencing of a fragment of the 18S rDNA showed only a 96% similarity with *B. canis*. A study on the prevalence and incidence of canine babesiosis in Western European countries, by means of questionnaires in veterinary clinics in 2010, did not yield any cases of canine babesiosis for Luxembourg [188]. However, a recent study reported the occurrence of the ornate dog tick, *D. reticulatus*, in the southern parts of Luxembourg ([189]; Figure 1).

### 3.6. Poland

#### 3.6.1. Babesiosis in Humans

In Poland, the first case of babesiosis, likely due to *B. microti*, was described as an imported infection in a 37-year-old immunocompetent sailor following his return from Brazil [190]. The first case of babesiosis in an immunosuppressed patient (a patient with ulcerative colitis, treated with immunosuppressive drugs) was reported in 2004 [191]. Although infection was confirmed by the successful cross-infection of mice, molecular identification of the *Babesia* species involved was unsuccessful [191]. No new cases were reported until 2010, when an asymptomatic infection with *B. venatorum*/*B. divergens* was identified by PCR in an immunocompetent forester from southeastern Poland [192]. In 2015, an asymptomatic infection with *B. microti*, Jena strain, was identified in two immunocompetent foresters from Białowieża, northeastern Poland (2 were positive out of 58 tested, 3.3% [193]). In 2016, another *B. microti* (identical to the pathogenic US Jena strains) infection was diagnosed in a hospitalized immunocompetent woman, following her return from the US and Canada (likely an imported case). The woman was co-infected with Lyme borreliae [194].

In addition to these sporadic cases, four epidemiological studies have been conducted in northeastern Poland, a region that is well known as (hyper-)endemic for other tick-borne diseases, including borreliosis (Lyme disease), anaplasmosis, and tick-borne encephalitis (TBE) [195,196,197,198]. In the first study, anti-*B. microti* IgG were found in five healthy foresters (5/114 = 4.4%) from Białowieża (local seroprevalence: 9.2%) [199]. In a second, larger study, which focused on *Babesia* infections in patients hospitalized/treated because of non-specific symptoms (fever, muscle pain, joint pain, headache, vertigo, nausea, and vomiting) following a tick bite, 548 patients were tested by a range of methods, including molecular methods (PCR and sequencing) and serology [200]. *Babesia* infection was diagnosed by PCR (6) and serology (3) in six patients (about 1%). Analyses of the obtained sequences revealed infection with a *B. microti* variant identical to the Munich strain (or Omsk-vole110 or UR2), a grouping that is considered to be non-pathogenic in humans, comprising isolates derived from rodents, mainly voles (*Microtus* spp.) [200]. In a study of 110 patients diagnosed initially with TBE, one patient (0.9%) tested positive for *B. microti* (by PCR and sequencing [201]. In another study performed by the same group, one of 118 patients (0.8%) with non-specific symptoms following a tick bite was diagnosed with co-infection with *Anaplasma phagocytophilum* and a *Babesia* sp. (undetermined species and strain) [202].

In a recent serological study focusing on the detection of tick-borne pathogens in HIV-positive patients and blood donors, anti-*B. microti* IgM was detected in 9.3% of HIV-infected patients (21/227) and in 1.0% of blood donors (2/199) [203]. Anti-*B. microti* IgG was detected in 2.2% of HIV-infected patients (5/227) and in 1.5% of blood donors (3/199) [203].

To summarize, both imported and autochthonous, asymptomatic, and symptomatic infections with *B. microti* have been reported in humans in Poland. Most of the positive cases have been observed in immunocompetent subjects. Interestingly, infections were caused both by well-known zoonotic *B. microti* strains (US or Jena or Gray) or by strains considered to be non-zoonotic, such as the Munich strain (or UR2 or Omsk-vole110) [193,194,200]. Additionally, an asymptomatic human infection with *B. venatorum*/*B. divergens* has also been identified [192]. Serological studies have revealed a relatively high seroprevalence of anti-*B. microti* antibodies in groups at risk (about 9% in foresters and HIV-infected patients).

#### 3.6.2. Babesiosis in Animals

Cases of babesiosis in animals other than dogs are rarely reported in Poland. There is a single published case of feline babesiosis reported to date: a 10-year-old cat, showing weakness, anemia, fever, and hematuria, which recovered fully after the administration of imidocarb [204,205]. Piroplasms in the blood smear of this animal resembled *B. canis*, but the sequencing of a fragment of the 18S rDNA showed only 95% homology with *B. canis*. However, in central Poland, a region that is hyperendemic for *B. canis*, cases of babesiosis in cats are suspected to occur sporadically: 2–3 cases per year, in comparison to an incidence of 240/1000 dogs at the same veterinary clinic [206].

There are few reports of *Babesia* spp. infections in cattle and horses, although several cases of bovine piroplasmosis have been diagnosed, including fatal cases, likely due to *B. divergens* in northwestern Poland (Choszczno) in 2017 and 2018 [207]. In eastern Poland (Lubelskie province) *Babesia* spp. DNA has been detected in 10.4% of apparently healthy dairy cows (20/192), with no effect on the hematological and biochemical parameters or milk productivity in the positive animals [208,209]. The exact identification of the piroplasm species involved was not possible because the sequencing of 18S rDNA revealed only a low similarity (max 93% identity) to *B. occultans*.

There are few cases of piroplasmosis in horses reported from Poland [210,211]. The first case was described in 2008, in a 2-year-old stallion with fever, anemia, loss of appetite, and muscle weakness. PCR sequencing revealed that infection was caused by *T. equi* [210]. Similarly, in the most recent study, the presence of *T. equi* DNA (identity above > 99.5%) was detected in 37 out of 512 (7.2%) horses with clinical signs following tick bites [211]. No *Babesia* infections were noted among these horses, which presented with lethargy, anemia, and thrombocytopenia [211].

Babesiosis in dogs is an increasing veterinary problem in large areas of Poland and is strictly associated with the occurrence of *D. reticulatus* ticks [212]. Before 2000, canine babesiosis was limited to the eastern region of the country [213]. However, with the expansion of the range of *D. reticulatus* toward west, central and eastern Poland, these regions currently constitute a large (hyper-)endemic area for canine *Babesia* infections [212,214,215], with thousands of canine babesiosis cases treated annually [210,212,216,217,218]. Interestingly, a new population of ornate dog ticks has been found in western Poland [219,220], but no *B. canis* DNA was detected to date in about 2100 examined ticks from this area [212,220]. In a recent country-wide epidemiological study, the incidence of canine babesiosis was calculated for different regions, based on the number of dogs treated for babesiosis by veterinary practitioners in 2018 [212]. The overall annual incidence of clinical babesiosis among the Polish dog population was 20/1000 dogs (2%), with marked differences between three regions of the country. The study revealed few cases and low incidence in western Poland (a total of 19 cases/year and 0.4/1000 dogs) and in the “gap” region where no *D. reticulatus* has been found to date (only 7 cases/year and 0.9/1000 dogs). Many more cases (1532) and a much higher incidence (53/1000 dogs), accompanied by 2.5% fatality, have been reported in the areas of central and eastern Poland. In an earlier study [217], the number of canine babesiosis cases was six times higher in dogs in the eastern regions of Poland compared to the western regions. Canine babesiosis is currently the most common cause for renal replacement therapy in dogs [221]. *Babesia canis* is reported almost exclusively as the etiological agent of canine babesiosis in Poland [214,215,218,222,223], with only four cases of *B. gibsoni* identified recently [224,225].

### 3.7. Slovakia

#### 3.7.1. Babesiosis in Humans

In a recent review [226] on ticks and tick-borne disease occurrences in Slovakia, three unpublished cases of human babesiosis are mentioned. No more data (i.e., species involved, imported vs. autochthonous, or the region of the country) has been provided to date.

#### 3.7.2. Babesiosis in Animals

No reports of babesiosis in cattle or cats have been published over the past 20 years, and no horses were found to be positive among 39 from southern and southwestern Slovakia, as examined recently by PCR for *Babesia* and *Theileria* [227].

Canine babesiosis is an emerging disease in Slovakia. Two studies describing the first recognized cases of babesiosis were published in 2001 [228] and 2002 [229], respectively. Swan et al. [228] described three cases of clinical babesiosis diagnosed in southwestern Slovakia (near Bratislava) in early autumn 2000. The infection was confirmed by the presence of *Babesia* sp. in erythrocytes, observed by microscopic examination of the blood of the dogs (one crossbreed, two Irish Setters). The outcome of the infection was fatal in the case of a female Irish Setter. Chandoga et al. [229] reported the first case of canine babesiosis in southeastern Slovakia, near Kosice, in a 1.5-year-old male Siberian husky in May 2000. In autumn 2000 and February 2001, two additional cases were diagnosed in this region [229]. Then, *B. canis* was found in 1% of *D. reticulatus* ticks in southwestern Slovakia in 2002 [230]. During an outbreak of babesiosis in the period from 2004 to 2005 and until 2010, veterinary practitioners from areas with the endemic occurrence of canine babesiosis in southern Slovakia collected 87 samples from dogs with clinical signs of babesiosis, and molecular techniques (PCR and sequencing) confirmed *B. canis* infection in 80/87 dogs [231]. In the same area, 326 (204 females and 122 males) questing adult *D. reticulatus* ticks were collected by flagging. The DNA of *B. canis* was identified in 35.6% of the ticks. Furthermore, *D. reticulatus* ticks were observed across the entire territory of Slovakia but were seen less frequently in northern areas of the country [232].

Marked west-to-east differences have been observed in the prevalence of *B. canis* in *D. reticulatus* ticks collected in Slovakia [233]. The highest prevalence of *B. canis* has been observed in *D. reticulatus* from eastern Slovakia (14.7%), whereas the prevalence in the southeastern region was significantly lower (2.3%). Notably, all 874 *D. reticulatus* ticks collected in western Slovakia (Záhorská Nízina lowland) tested *B. canis*-negative. In a more recent study (2010–2011), a group of 217 dogs, encompassing both healthy individuals and those treated for babesiosis, was tested both by molecular techniques and serology [234]. The dogs originated from western (near Bratislava) and southern Slovakia (near Nové Zámky and Komarno). Interestingly, no dogs from the western region tested positive for *B. canis* (including dogs with suspected babesiosis), in comparison to 31–35% of (sero-)positive dogs from southern Slovakia [234]. Thus, the authors concluded that canine babesiosis is surely endemic in southeastern and southern Slovakia but may not yet be endemic in western regions of the country, despite the recent expansion of the parasite in a northwestern direction ([234], Siroky, unpublished).

In 2013, two pit bull terriers from one household, both with clinical signs of babesiosis, were treated with imidocarb without success. Molecular testing revealed *B. gibsoni* infection in both dogs [106], despite the absence of the main tick vector, *R. sanguineus*, in Slovakia.

### 3.8. Slovenia

#### 3.8.1. Babesiosis in Humans

Testing of sera from seven febrile patients with a history of tick bites by an “in-house” IFAT [230] resulted in the detection of anti-*B. divergens* antibodies in five patients. However, molecular testing revealed negative results for these patients and they also tested negative in an anti-*B. microti* IFAT.

In 2014, a *B. crassa*–like infection was diagnosed by a range of methods, including molecular testing (PCR and sequencing) in a 55-year-old asplenic woman [235]. The patient sought medical treatment after a 6-day history of intermittent fever up to 39 °C, myalgia, headache, poor appetite concomitant with weight loss, fatigue, sweating, and dark urine. She reported no history of travel, tick bites, animal contact, or blood transfusions.

#### 3.8.2. Babesiosis in Animals

There are only very limited data on babesiosis in domestic animals from Slovenia. In the period from 2000 to 2002, based on clinical, microscopic, and molecular investigations, 14 (5.9%) of 238 dogs admitted to the animal clinic in Ljubljana were infected with *Babesia* spp. Clinical signs relating to acute hemolysis, fever, anorexia, depression, and hematological abnormalities such as anemia and thrombocytopenia were noticed in the majority of the 14 infected dogs. *Babesia canis* was detected in 11 dogs (4.6%) and *B. vogeli* in 3 dogs (1.3%) via PCR and sequencing [236].

Spleen samples from 51 roe deer (*Capreolus capreolus*) and 30 red deer (*Cervus elaphus*) were tested for piroplasms between 1996 and 2000. Both *B. divergens* and *B. venatorum* were detected in roe deer (76.5%); however, more animals were infected with *B. divergens* (54.9%) than *B. venatorum* (21.6%). Only 16.7% of red deer tested positive for *B. divergens* alone [237]. Additionally, *B. venatorum* DNA was detected in *I. ricinus* ticks from Slovenia [238].

### 3.9. Switzerland

#### 3.9.1. Babesiosis in Humans

Overall, reports on human babesiosis in Switzerland are rare, despite a high incidence of other tick-borne infections [239]. There are only three clinical human case studies in Switzerland in the last 30 years: one was due to an autochthonous infection with *B. microti*, one was a *B. microti* infection in a tourist from the eastern United States, and one was an imported case tested positive for *B. divergens,* in a patient returning from Wales [240,241,242]. All patients had no history of immunosuppression and showed only a mild course of the infection with fever. Interestingly, in a region with 4% *B. microti* PCR-positive questing *I. ricinus* ticks, a seroprevalence of 1.5% has been found by a *B. microti* IFAT in 396 residents [243].

#### 3.9.2. Babesiosis in Animals

Local outbreaks of babesiosis in cattle have been described in the past in different regions throughout Switzerland, including the western, central, alpine, and southern parts of the country [244]. These cases were due to small *Babesia* sp., most probably *B. divergens*, as this species could be identified in the tick vectors [245,246]. Sporadic *Babesia* outbreaks in farms are known to occur in the region of the Jura mountains (close to the Swiss-French border) up to the geographical tripoint where the borders of Switzerland, France, and Germany meet, and enzootic stability in the affected farms is suspected. Accordingly, a study indicated 90% seropositivity with antibodies against *B. divergens* in cattle in this region, but only a few (<1%) clinical cases were observed [247].

The presence of large *Babesia* species in cattle was identified in the southern part of Switzerland, and this was assumed to be *B. major*, based on the presence of the tick vector *H. punctata* [244]. More recently, the presence of *B. major* has been confirmed by PCR in ticks from the region [244].

Furthermore, a severe outbreak was reported on a trading farm for dairy cows in eastern Switzerland, with 3% (*n* = 8) of all examined animals testing positive for large *Babesia* sp. [248]. Species-specific *Babesia* sequencing for the positive samples was achieved at a later stage, with 99.7% sequence identity to *B. bigemina* [246]. Interestingly, 90% of the animals in this farm had up to five other tick-borne infections, presenting also with *Theileria* sp., *Anaplasma marginale*, *A. phagocytophilum* and *Mycoplasma wenyonii*. The *Theileria* sp. belonged to the complex of *T. buffeli*/*orientalis/sergenti*, which are considered to be less pathogenic and are disseminated throughout southern Europe [249,250].

Equine piroplasmosis is considered a sporadic disease in Switzerland but is estimated to be highly underdiagnosed [251]. The first description of EP in Switzerland was from 1985, when a stable with sports horses had an extensive outbreak [252]. Although some of the infected horses had traveled to sports events in other parts of Europe (mainly to France), most of the infected animals had no history of travel. Furthermore, autochthonous cases of tick-transmitted or iatrogenic infections in horses have been described in Switzerland [253]. An overall seroprevalence of 7.3% for EP was demonstrated in 689 horses, with a significantly higher seroprevalence of *T. equi* infections (5.8%) compared to *B. caballi* (2.9%), among which 10 horses (1.5%) were seropositive for both parasites. In imported horses, the seroprevalence of *T. equi* was significantly higher than in indigenous horses [254]. Between 2006 and 2016, 29 horses were presented at clinics for equine medicine at the Vetsuisse Faculty of the University of Zurich, with a diagnosis of EP [255]. The majority (62.1%) had a confirmed infection with *T. equi*, 34.5% with *B. caballi*, and one horse showed a co-infection with both species. Most of these animals (22 of 29) had a travel history to another country prior to the onset of symptoms. Most affected stables with autochthonous infections were located in the Jura mountains region, where tick vectors are present. Altogether, EP is prevalent in Switzerland, with sporadic outbreaks in regions where the tick vector is present, and constant monitoring is recommended [254].

The regional distribution of canine babesiosis in Switzerland is similar to the epidemiological pattern of EP. The first cases of *B. canis*, as the only large *Babesia* species prevalent in Switzerland, were described in 1974 in the region of Geneva [256]. Since then, a stable endemic focus has been present in the western part of Lake Geneva and along the region of the Jura mountains [257,258]. Sporadic outbreaks with severe infections in dogs without any travel history have been identified in eastern areas of the Swiss midlands and in northeastern Switzerland, where tick vectors (*D. reticulatus*) were found by flagging for questing ticks [26,259,260,261]. In these studies, 10 of the 50 dogs that had been admitted to veterinary practices between 2003 and 2015 died despite the initiation of treatment. After a few years, no further infections were observed and no endemic regions have been established so far, other than the one bordering France.

To our knowledge, there are no reports of autochthonous infections with small *Babesia* species (e.g., *B. gibsoni* and *B. felis*) in dogs and cats in Switzerland. In cats, infections with another apicomplexan tick-borne hemoprotozoan, *Cytauxzoon* spp., emerged, with a report in 2018 of five domestic kittens from the northwest and west of Switzerland presenting with severe anemia [262]. Furthermore, six infected domestic cats were identified in 2019 in the central part of Switzerland, triggering a nationwide epidemiological study, in which one seropositive (1 of 881 randomly selected cats), and one PCR-positive cat (1 of 501 anemic cats), both originating from the region of the Jura mountains, were identified [263]. In summary, these cases in companion animals indicate a higher risk of hemoprotozoan infections in the western part of Switzerland, where also tick vectors such as *D. reticulatus* can frequently be encountered [25]. Furthermore, babesiosis still remains an important imported and travel disease for companion animals in Switzerland, as supported, for example, by a report of several vector-borne diseases in rescued dogs imported from Hungary, which included two cases (15.4%) of *B. canis* [264].

Three *Babesia* species involved in human infections, *B. divergens*, *B. venatorum*, and *B. microti,* have been described in ticks from urban and suburban areas throughout Switzerland, representing a potential risk of infection for local inhabitants [243,245,246,247,265,266]. In individual studies, species identification has been carried out, and this has indicated a low prevalence of questing ticks (mainly *I. ricinus*), with 0.2–0.8% testing positive for *B. divergens*, 0.4–2.0% for *B. venatorum*, and 0.2–0.7% for *B. microti* [245,246,266]. In urban areas, *B. venatorum* has been detected only in areas with a known presence of wild ungulates [267].

## 4. Northern and Northeastern Europe

### 4.1. Denmark

#### 4.1.1. Babesiosis in Humans

The first case of human babesiosis in Denmark was an imported case of *B. microti* infection from the US, reported in 2013 [268]. No locally acquired cases have been reported to date.

#### 4.1.2. Babesiosis in Animals

Bovine babesiosis is endemic in Denmark but has received relatively little attention [269]. In 2018, the first autochthonous case of canine babesiosis was diagnosed in a Golden Retriever with no history of travel or import [270]. Earlier, a fatal case was reported in a Miniature Schnauzer that had traveled to Hungary [271]. In February 2017, 21 adult male *D. reticulatus* ticks were found on a migrating golden jackal (*Canis aureus*) that had been hunted in Western Denmark, about 200 km north of the Denmark–Germany border [272].

*Babesia microti* and *B. venatorum* were reported in ticks collected from domestic dogs in 2011 in Denmark [273], and *B. venatorum* in ticks from raccoon dogs (*Nyctereutes procyonoides*) [274].

### 4.2. Estonia

#### 4.2.1. Babesiosis in Humans

No published data are available from Estonia on babesiosis in humans.

#### 4.2.2. Babesiosis in Animals

There is still a paucity of information on the current situation regarding babesiosis in animals in Estonia because, thus far, no large studies have been undertaken among different animal species. According to old records from the period 1960–1968, bovine babesiosis was then estimated to affect 0.36% of cattle in the Estonian Soviet Socialistic Republic [275].

According to the Estonian Veterinary and Food Laboratory’s yearly report on animal diseases in 2020, 42 blood samples from horses were tested for EP and 14 (33.3%) were positive for piroplasms (Estonian Veterinary and Food Laboratory, 2020).

There have been two case studies published on canine babesiosis relating to Estonia. In 2010, six dogs attended a race meeting in Estonia. After their return to Poland, two of the dogs developed clinical signs of babesiosis [276]. *Babesia* DNA was detected in their blood samples and sequenced. Based on a phylogenetic tree, no regional specificity of the parasites was observed; therefore, the origin of the infection in the two cases could not be clearly determined. High homology to genotype 2 and an isolate from *D. reticulatus* from Kury, Poland, suggested that the dogs had been infected in Poland [276].

In 2015, a previously splenectomized dog was admitted to a small animal clinic of the Estonian University of Life Sciences in Tartu, Estonia, showing signs of lethargy, a change in urine color, and lack of appetite, was diagnosed with babesiosis by blood smear microscopy [277]. The clinical status of the dog worsened within 8 h, with the onset of unresponsive seizures; therefore, with the owner’s consent, the dog was euthanized. Blood samples from the dog and from two other dogs from the same household tested positive for *Babesia* using molecular methods, and the sequencing of the 18S rRNA gene fragment confirmed the causative species as *B. canis*. All the dogs had attended a dog show in the United Kingdom and clinical signs developed 13 days after their participation in the show; thus, it was considered likely that the infection had been acquired abroad. This case study is a useful reminder of the possible rapid progression of the disease in splenectomized dogs [277].

Various zoonotic *Babesia* species, for example, *B. microti*, *B. divergens* and *B. venatorum,* have been identified in ticks from Estonia [278].

### 4.3. Finland

#### 4.3.1. Babesiosis in Humans

There is one published case study of fatal human babesiosis caused by *B. divergens*, in a 53-year-old man with a rudimentary spleen and comorbidities [279]. The man had no travel history in the previous five years and the infection was considered to have been acquired from a local cattle reservoir through a tick bite [279].

#### 4.3.2. Babesiosis in Animals

Bovine babesiosis is endemic in Finland [279,280] although, currently, cases are reported to be far less common than in the 1960s, when thousands of cases were reported annually [281]. The most recent case of EP was reported in 2020 in an imported horse [280]. Canine babesiosis due to *B. canis* has been diagnosed in imported dogs [282,283]. There are no published reports of the endemic occurrence of *D. reticulatus* in Finland (ECDC website, Figure 1).

### 4.4. Iceland

Neither imported nor autochthonous cases of babesiosis in humans or animals have been reported from Iceland. No endemic tick populations have been detected in the country (ECDC website, Figure 1).

### 4.5. Latvia

#### 4.5.1. Babesiosis in Humans

There are no published reports of human babesiosis in Latvia to date.

#### 4.5.2. Babesiosis in Animals

No cases in cats or horses in Latvia have been reported. The first documented cases of babesiosis in dogs without a travel history occurred in Latvia between 2009 and 2011 [284]. Since then, canine babesiosis has become an endemic disease in the southern and western regions of Latvia and is caused solely by the large species, *B. canis* [285]. A seasonal pattern has been observed in Latvia for disease outbreaks, as the majority of canine babesiosis cases have occurred between April and June [285]. The distribution of *B. canis* in Latvia is most likely linked primarily to the expansion of the range of the main vector—*D. reticulatus*. A recent study has shown that *D. reticulatus* ticks are present in the southern, central, and western regions of the country; moreover, this tick has been detected in geographically separate small localities in the Riga region, outside of the major region of endemicity [286]. In accordance with this finding, many canine babesiosis cases have been reported from Riga, the capital of Latvia [285]. In recent assessments, the reported overall prevalence of *B. canis* in field-collected *D. reticulatus* ticks in Latvia was found to be relatively low (0.34%); in contrast, 14.8% of *D. reticulatus* ticks removed from Latvian dogs were *B. canis*-positive [286,287]. Additionally, *B. canis* DNA was detected in 0.91% of the field-collected *I. ricinus* ticks; however, *B. canis*-positive samples were found only in areas with the sympatric occurrence of *I. ricinus* and *D. reticulatus* [286,288]. Several *Babesia* species have been identified in field-collected ticks, including the human pathogens *B. microti* and *B. venatorum* [286,288].

### 4.6. Lithuania

#### 4.6.1. Babesiosis in Humans

In Lithuania, babesiosis is not a nationally notifiable disease and no human cases have been reported.

#### 4.6.2. Babesiosis in Animals

Confirmed babesiosis cases among domestic animals have been reported only in dogs. There are no reports of *Babesia* infection in cats, cattle, or horses in Lithuania. However, during observations of the health status of European bison (*Bison bonasus*) in Lithuania, *B. divergens* was detected in two out of 37 (5.4%) spleen samples from these animals and in an engorged *I. ricinus* feeding on the bison. These findings were based only on molecular evidence (PCR and sequence analysis). However, visual examination of the internal organs of the animals revealed pathological changes that were compatible with those expected of *Babesia* infection [289]. The detection of *B. divergens* in European bison and *I. ricinus* ticks in Lithuania implies that domestic livestock coexisting with European bison are at high risk of contracting this pathogen.

The first cases of canine babesiosis were recorded and confirmed by microscopic analysis in central Lithuania in the early 2000s. Although previously a rare infection in the country, canine babesiosis has been reported more frequently in recent years in Lithuania. According to the observations of veterinary practitioners in the period from 2003 to 2010, the highest incidence of canine babesiosis was observed at the time in the central and southwestern parts of Lithuania [290]. However, over the last decade, canine babesiosis has been spreading continuously in Lithuania and an increasing number of cases, with a wide variety of clinical signs, have been recorded throughout the country. Currently, the disease occurs almost across the entire country. Both complicated and uncomplicated forms of canine babesiosis have been recorded [291,292].

*Babesia canis* is reported as the main etiological agent of the disease in dogs in Lithuania [290,291,292]. The expansion of the range of *B. canis* in Lithuania is directly related to the expanding range of the main vector—*D. reticulatus*. Evidence for the changing distribution of *D. reticulatus* ticks in the Baltic countries has been provided by the occurrence of canine babesiosis in new locations in Lithuania and Latvia since 2013 [284]. In Lithuania, the presence of *D. reticulatus* (2013–2015) has been confirmed in 38 localities in which this species had not been previously reported [293]. *Babesia canis* DNA was detected in 1.2% (26 of 2255) of *D. reticulatus* ticks collected from 40 locations across Lithuania. The highest prevalence of infection was observed in the central (7%) and southern (11%) parts of the country, where populations of *D. reticulatus* ticks have been observed since the last century and dogs are frequently diagnosed with canine babesiosis [294].

The State Food and Veterinary Service of Lithuania does not hold statistics on the overall annual incidence of clinical babesiosis cases, nor does it record mortality among the Lithuanian dog population. From 2011 to 2018, veterinary practitioners from different small animal clinics in central (Kaunas), eastern (Vilnius), and western (Klaipeda) parts of Lithuania reported between 20 and 150 babesiosis cases per year, with the highest number of cases detected in central regions of the country. According to information provided by the major veterinary clinic in Central Lithuania (Dr. L Kriaučeliūnas Small Animal Clinic; Faculty of Veterinary Medicine, Lithuanian University of Health Sciences, Kaunas), the incidence of canine babesiosis (based on the number of treated babesiosis cases) in 2019 was 59 cases/year and 8.6/1000 dogs; in 2020, the figure was 74 cases/year and 10.5/1000 dogs; and in 2021, it was 55 cases/year and 7.1/1000 dogs (Radzijevskaja et al., unpublished). During the past few years, some veterinary practitioners have noticed a trend of decreasing canine babesiosis cases, especially of the complicated forms of the disease [291], most likely attributable to the increasing public availability of information and the implementation of measures for the prevention of canine babesiosis.

The causative agents of human babesiosis *B. divergens*, *B. venatorum* and *B. microti* have been detected in *I. ricinus* and *D. reticulatus* tick populations in the country [289,294,295].

### 4.7. Norway

#### 4.7.1. Babesiosis in Humans

The first case of severe autochthonous babesiosis in Norway was reported to have occurred in 2007 [296]. The patient was a splenectomized 58-year-old male veterinarian who worked with cattle. He was frequently exposed to tick bites in an area endemic for bovine babesiosis in western Norway [297,298]. The patient presented with severe hemolysis and multi-organ failure. Microscopic observation revealed a 30% parasitemia with a small *Babesia*, and IFAT confirmed a *B. divergens* infection [296].

In a study of 188 patients with erythema migrans, no *Babesia* DNA was detected in skin biopsies, nor were anti-*B. microti* antibodies detected in the serum samples by IFAT [299]. No *Babesia* DNA was detected in blood samples from 163 immunosuppressed patients with systemic rheumatic, gastrointestinal, and neurological autoimmune diseases from southern Norway [300]. In contrast to these findings, among the adult general population in Søgne in the southernmost part of Norway, IgG antibodies to *B. microti* were found in 2.1% (33/1537) of serum samples [301].

#### 4.7.2. Babesiosis in Animals

Norway is endemic for bovine babesiosis, with a regular occurrence of cases [297,298,302]. Records of the tick-borne disease, bovine babesiosis, are available from the Norwegian Cattle Health Recording system (NCHRS) for the time period from 2006 to 2015 [297,298,302]. The registration is based on clinical symptoms, rather than confirmed cases based on laboratory diagnostics. Records of babesiosis in cattle are rare in eastern Norway but are regularly reported in the western regions of the country, with incidence values ranging from 5 to 30/1000 [297,298].

Until recently, canine babesiosis caused by *B. canis* was considered to be an imported disease; imported cases of acute babesiosis were diagnosed sporadically in dogs returning from Central Europe (Brun-Hansen and Øines, unpublished). The first likely autochthonous case was diagnosed in early May 2009, in an Irish Setter from the Oslo area [303]. The 6-year-old male dog presented at a veterinary clinic with lethargy, anorexia, and polydipsia. The owner had noticed the dog becoming sick one day after a large (about 8–10 mm) gray tick had been removed from its neck. Unfortunately, the tick was not available for species identification or further investigation. Babesiosis due to *B. canis* was diagnosed, based on microscopy and molecular methods (PCR and sequencing). There are no data on the occurrence/distribution of *D. reticulatus* in Norway (Figure 1).

### 4.8. Sweden

#### 4.8.1. Babesiosis in Humans

The literature records two human cases of babesiosis in Sweden to date. The first report was a 34-year-old splenectomized man who presented in October 1989 with fever, myalgia, and dysuria [304]. His condition rapidly deteriorated as he became anuric and developed severe hemolytic anemia, thrombocytopenia, and fibrinolysis. Peripheral blood smears revealed 40% parasitemia with *B. divergens* [304]. The second case was of a 52-year-old splenectomized man, who presented with recurrent fever between March and May 2015, muscle pain, and dark urine [305]. *Babesia* sp. infection was diagnosed, based on microscopy, and *B. venatorum* infection was identified by PCR and sequencing [305]. An epidemiological study from southern Sweden estimated the seroprevalence of IgG antibodies to *B. microti*/*B. divergens* to be 2.5% (5/197) among healthy individuals and 16.3% (14/86) among individuals seropositive for *Borrelia burgdorferi* s.l. antibodies [306]. In a recent study, the seroprevalence of antibodies to *B. microti* was 4.4% (7/159) among individuals bitten by ticks [307].

#### 4.8.2. Babesiosis in Animals

Bovine and canine babesiosis are notifiable animal diseases in Sweden [308]. Bovine babesiosis is endemic in Sweden [309,310] although no cases were recorded in 2019 [308]. Canine babesiosis is sporadically diagnosed as an imported disease. Four cases of babesiosis due to *B. canis* infection and one case due to *B. gibsoni* infection were reported in 2019 [308]. No cases of feline or equine babesiosis were published or recorded.

## 5. Discussion

A thorough review of the epidemiological situation in the range of countries covered in this paper revealed an interesting picture. First, cases of human babesiosis have occurred in almost all regions of Europe (in 13/20 of the reviewed countries) and their number is slowly but steadily growing. In addition to the well-known cause of human babesiosis in Europe, *B. divergens*, there are increasing numbers of cases/infections attributable to other species, i.e., *B. venatorum*, *B. crassa*-like, and *B. microti*. Furthermore, infections due to the last species seem to be more prevalent than generally believed, as is indicated by the frequent detection of antibodies to *B. microti* in different European populations and the detection of *B. microti* DNA in patients from different countries (Austria, Germany, Hungary, the Czech Republic, Switzerland, and Poland) [3]. A recent study has reported that the molecular detection of *B. microti* enabled convincing identification of this parasite as the etiological cause of non-specific symptoms leading to patients’ hospitalization in northeastern Poland [200]. The study also provided evidence that a strain of *B. microti*, common in voles (*B. microti* Munich) [7] and regarded as non-zoonotic, can lead to the clinical manifestation of the infection in humans [200]. Moreover, the study carries an important message, i.e., that babesiosis due to *B. microti* may be easily overlooked and may not be recognized in immunocompetent patients due to the non-specific symptoms and to the generally low awareness of general practitioners regarding this tick-borne zoonosis. There is a clear need not only to standardize diagnostic tests and to employ antigens from European *B. microti* strains in tests but also to develop tests for the detection of antibodies against *B. divergens* and *B. crassa*- like organisms.

In Western Europe, human babesiosis is rare but cases due to *B. divergens* and *B. microti* (autochthonous or imported) have been regularly reported [3]. In France, 21 cases have been described to date, including 19 cases due to *B. divergens* and two cases due to *B. microti* (imported from the US) [3]. In the British Isles, nine cases have been reported to date, including eight cases due to *B. divergens* (7 in the UK and one in Ireland) and one imported case due to *B. microti* [3]. In southern Europe, ten cases have been reported in Spain (5 × *B. divergens*, 5 × *B. microti*, including four likely imported cases of the latter) [3]. The number of described human babesiosis cases from eastern Europe is much lower, despite the fact that TBD (mainly Lyme disease, TBE, or rickettsioses) constitute a serious health problem in this region [311,312]. There have been at least two cases from Russia [3,313,314], both due to *B. divergens*. No human cases have been reported from Belarus to date. No human cases have been reported from Ukraine; however, in a recent serological study from Kharkiv, antibodies to *B. divergens* (6.9%) and *B. microti* (3.4%) were detected, with higher frequency in HIV-infected individuals (26.7%) and in Lyme disease patients (16.7%) than in blood donors (1.7%) [315].

Co-infection of *Babesia* spp. and *Borrelia burgdorferi* s.l., as well as *Babesia* spp. and *A. phagocytophilum,* or TBE have been reported in patients from the reviewed countries.

In view of the present emergence of human babesiosis in the US, Canada, China, and other countries outside Europe [5,16], and the emergence of other tick-borne diseases in Europe, a very likely scenario for the next decade is a more rapid increase in the cases of babesiosis, especially in the central and eastern regions of Europe, as reviewed herein. Among animal babesiosis, canine babesiosis is a disease of significant impact, both in well-known endemic (Central Europe) or newly identified (Northeastern Europe, including the Baltic states) regions. Interestingly, the occurrence of (hyper)endemic foci/regions of babesiosis due to *B. canis* is rather patchy/mosaic and depends on the occurrence of tick vector populations, which are not evenly distributed across the terrain of most countries [23,24,25,316]. Although the ECDC recognized the great majority of the reviewed countries as being endemic for *D. reticulatus* (Figure 1), the exact distribution rarely covers the whole territory of a specific country. Nevertheless, *B. canis* is well established in the endemic areas/spots of Austria, BiH, Croatia, Serbia, Hungary, Poland, Slovenia, Slovakia, Switzerland, Latvia, and Lithuania, and newly endemic foci have appeared in many other countries, for example, Germany and the Czech Republic. Overall, autochthonous *B. canis* infections have been reported from 15 out of 20 reviewed countries. Although in some countries (e.g., in Switzerland and Austria) the number of cases appears sporadic but stable, in other Central European countries, babesiosis due to *B. canis* is an emerging and fast-spreading disease (Supplementary Table S1). Nordic countries are an example, a region where the spread to new areas is currently being observed, with the first autochthonous published cases of *B. canis* infections in dogs from Norway and Denmark [270,303]. The occurrence of sporadic cases, together with reports on *D. reticulatus* ticks, for example, near Stockholm or in other southern parts of Scandinavia, constitute the first steps for the development of local endemicity [272,303]. Furthermore, marked differences in terms of protective practices against ticks have been identified in the Nordic countries [317].

A questionnaire study reported that 23% of veterinarians working in the Baltic and Nordic countries saw at least one canine babesiosis case in 2016 [318]. A veterinarian working in the Baltic countries had 12.2 times higher odds of seeing a canine babesiosis case than a veterinarian working in the Nordic countries. Almost half (48%) of the veterinarians responding to the survey answered correctly that canine babesiosis is not considered a zoonosis [318].

The emergence of canine babesiosis due to *B. canis* is closely connected to the expansion of the range of *D. reticulatus* [22,97,212,319]. Countries in which the occurrence of *D. reticulatus* has been confirmed are shown in Figure 1. However, there are marked differences in the prevalence of *B. canis* in *D. reticulatus* from different parts of central Europe, with generally higher prevalence in the eastern European tick populations [220,294,316,320,321]. There are a few reports of very low prevalence, at below 1% and even zero [97,322], among the western European tick populations, although sporadically very high prevalence has also been observed, notably in novel local foci, such as those reported in the UK and Switzerland [26,319]. The prevalence of *B. canis* in ornate tick populations from the different areas of a single country may differ markedly, as observed in the Czech Republic, Slovakia, and Poland [97,233,316]. Accordingly, the highest incidence of canine babesiosis (from 5 to 250 cases/1000 dogs) is reported from areas with a high percentage of infected ticks—i.e., in eastern Poland, western Ukraine or southwestern Lithuania [212,294,320,323]. In the future, it will be important to monitor carefully whether these differences in the prevalence of *B. canis* between tick foci/populations are maintained, or if prevalence changes with further increases in tick density/population/range.

Besides *B. canis*, several other species of piroplasms, such as *B. vogeli*, *B. gibsoni*, *B. vulpes*, *B. caballi,* and *T. equi* [40], have been identified in dogs and, recently, *T. capreoli* was confirmed by PCR and sequencing as the cause of the septic shock and death of an infected dog [44]. In some regions, the fatality rate in dogs from *Babesia*/*Theileria* infection could be higher than suspected, since post-mortem molecular detection has not been performed on a regular basis and infections may have been misdiagnosed, possibly being confused with *A. phagocytophilum, Borrelia burgdorferi* s.l., *Leptospira* infection [44] or intoxication. Furthermore, due to the rapid clearance of merozoites after treatment in cases with a history of anti-babesial treatment, PCR diagnostic testing should be used rather than the microscopical examination of blood smears, since it is virtually impossible to detect any merozoites on slides within 24 h after treatment [45].

Wild carnivores, such as grey wolves and red foxes, may play an important role as subclinical carriers of babesiosis, as well as vector tick species, and spread both to non-endemic regions [46,47,324,325]. In a recent study, a young collared and telemetrically followed grey wolf infected with *B. canis* roamed over a large area, covering 563 km^2^ during a 12-month period, and, throughout, may have constituted a source of *Babesia* infection for *D. reticulatus* ticks that it picked up during its travels in the region [46].

Feline babesiosis is still very rarely reported globally [12], including among the reviewed countries. Although there are several species of *Babesia* that can infect cats [12], these piroplasms have not been identified in questing *I. ricinus* or *D. reticulatus* (reviewed in [8]). Cats are less frequently infested with *D. reticulatus* than dogs [22,326], and their susceptibility to *B. canis* is still to be confirmed. Interestingly, in a hyper-endemic region for canine babesiosis in central Poland, sporadic feline cases are treated based on clinical symptoms that are indicative of babesiosis (Olszak, personal communication). Unfortunately, there have been no attempts to reach a species-level diagnosis in these cases.

Piroplasmosis in horses is reported regularly in several of the countries reviewed herein, and it is caused most frequently by *T. equi*. Again, the tick vectors in central and northeastern Europe occur focally (i.e., *H. punctata* in Germany) [116], or are not reported (there are no reports of *T. equi* in *D. reticulatus*; [8]). Therefore, many cases are likely to have been imported (i.e., to the Czech Republic, Germany or Finland) [123]. Moreover, because *Theileria* spp. are transmitted only transstadially (no transovarial transmission) in ticks, this precludes the establishment of endemic foci within tick populations in the absence of infected animals (source of infection for ticks). *Theileria equi* occurs commonly in southern Europe, where the tick species that act as vectors are mainly *R. sanguineus* s.s. or *Haemaphysalis* spp., in contrast to central and northern Europe, where *D. reticulatus* or other *Dermacentor* sp. or *Hyalomma* ticks may be involved. With climate change and the global movement of equids, it is likely that *Theileria* spp. will become more common in central Europe, as has recently been observed in Germany [27] and Hungary. In contrast, *B. caballi* infections, transmitted by *Dermacentor* and *Hyalomma* spp., seem still to be rare in the reviewed countries [27,211]. However, due to the transovarial transmission of *Babesia* spp. in ticks, the establishment of new endemic foci may increase in the future.

Nearly all the reviewed countries (16/20; Supplementary Table S1) have endemic areas for bovine babesiosis due to *B. divergens*. *Babesia divergens* is a zoonotic species and infections can be fatal in both cattle and humans [3,13]; thus, the occurrence of babesiosis due to *B. divergens* in cattle is of special relevance. Interestingly, in the last twenty years, the number of cases has decreased in parallel in both humans [3] and cattle [123,141], a trend that has been observed across several of the countries reviewed herein, this despite the repeated recent reports of the occurrence of this piroplasm in *I. ricinus* ticks over much of Europe [8,327]. The trend is usually explained by the less extensive use of pastures, anti-tick prophylaxis, and by the maintenance of immunity in parasite-exposed herds, leading to enzootic stability [123,141,328]. However, new outbreaks of bovine babesiosis have been observed recently, e.g., between 2017 and 2019, in northern Germany [123], BiH, and likely northwestern Poland (Supplementary Table S1). These outbreaks draw attention to bovine babesiosis as a potential re-emerging disease.

Despite variations in the methods applied in the studies covered by this review, precluding direct comparisons in many cases, the extensive epidemiological and molecular data collated suggest the expansion (and emergence) of new piroplasm species in central, northern, and northeastern Europe. For instance, sporadic cases of canine babesiosis due to *B. gibsoni* have been reported in Austria, the Czech Republic, Slovakia, Germany, Hungary, Poland, and Sweden (Supplementary Table S1), despite the lack of the main vector (*R. sanguineus*) in most of these countries. *Babesia gibsoni* infections could have been imported or transmitted by alternative routes, through dog fights/biting, or transplacentally [329,330]. Interestingly, almost all reported cases have been described in American pit bull terriers or American Staffordshire terriers, suggesting either the higher susceptibility of these breeds to piroplasms or specific but as yet unidentified transmission routes within these breeds. Furthermore, *B. vogeli* was identified in dogs from Croatia, Serbia, and Slovenia. In Hungary, *B. major*, *T. buffeli,* and *T. orientalis* were recently identified in cattle [168].

Considering the geographical occurrence of babesiosis in animals, the greatest diversity of piroplasms has been observed in southern Europe (Supplementary Table S1). Central and northeastern Europe (the Baltic states) are the main areas of expansion and the emergence of new foci for *Babesia*/*Theileria* species. Fennoscandia is endemic for *B. divergens,* enabled by the occurrence of *I. ricinus* ticks [21], but it seems to be largely free of the endemic occurrence of canine babesiosis and other *Babesia* spp. due to the lack of established tick vector populations. However, with the expected further, future, successful expansion of *D. reticulatus* in central and northeastern Europe ([22,212,293,294,331], Figure 1), and due to the transovarial and transstadial transmission of *Babesia* spp. within tick species, it is likely that areas of Finland and southern Scandinavia will become endemic for piroplasms vectored by this tick species in the relatively near future.

Undoubtedly, the situation in Europe is changing, but a clear picture of future trends is not easy to generate, given the data currently in the public domain and the lack of attention to human and animal babesiosis in some countries. Different sampling approaches and different methods have been applied, complicating a direct comparison between studies and the synthesis of a global picture. Increasing awareness about babesiosis in both the medical and veterinary professions, as well as among the general public, is urgently required. However, in order to enable the identification of convincing temporal changes and trends in the prevalence and associated disease status of babesiosis, we firmly recommend the standardization of data collection and the better coordination of studies across the countries, sectors, and disciplines involved.

## 6. Conclusions

This review of the occurrence of piroplasms in humans, animals, and ticks from 20 countries, covering southeastern, central, northern, and northeastern Europe, has revealed the widespread occurrence of these pathogens. Recorded cases of human babesiosis are still rare but their number is expected to rise in the coming years, due to the widespread and longer seasonal activity of *I. ricinus,* with more cases attributable to *B. microti* and because of better diagnostic methods. Bovine babesiosis has re-emerging potential because of the likely loss of herd immunity and the return to more extensive cattle management. Canine babesiosis due to *B. canis* is a rapidly expanding and emerging tick-borne disease in central and northeastern Europe, the prevalence of which correlates with the rapid and successful expansion of the ornate dog tick population. There is clear potential for the local establishment of new “hotspots” and, on the basis of currently identified trends, the focal emergence of disease in areas of Fennoscandia in the near future. In horses, *T. equi* infections appear to be spreading in central Europe, mainly due to the mobility of horses. Feline babesiosis is still reported only rarely in Europe but the susceptibility of cats to *B. canis* infection requires reassessment and their risk of infection needs re-evaluation, especially in those regions of Europe that are endemic for babesiosis.

Finally, in this review, we have drawn attention to the still largely incomplete datasets currently available about the extent of babesiosis in Europe. Despite this, there is clear evidence of an increasing annual incidence of piroplasmosis across Europe, which is changing in line with similar increases in the incidence of other tick-borne diseases that have been documented elsewhere (ECDC). This situation is of concern, and we recommend careful, standardized monitoring using a “One Health” approach.

## Data Availability

Not applicable.

## Supplementary Materials

The following supporting information can be downloaded at: https://www.mdpi.com/article/10.3390/microorganisms10050945/s1, Table S1: Babesiosis cases in humans and animals in the reviewed countries.
