# Supplementary material for: Babesiosis in Southeastern, Central and Northeastern Europe: An Emerging and Re-Emerging Tick-Borne Disease of Humans and Animals
Source: Microorganisms. 2022 Apr 30;10(5):945. doi: 10.3390/microorganisms10050945 (PMC9146636; doi:10.3390/microorganisms10050945)
Supplement: Supplementary file 1 [file microorganisms-10-00945-s001.zip › microorganisms-1667744-supplementary.pdf]

Supplementary Table S1

Babesiosis cases in humans and animals in the reviewed countries

Region and country	In humans	In dogs	In cats	In cattle	In horses
**SE Europe**					
BiH	nd	*B. canis* endemic, *Dr* expanding	nd	2 *B. divergens* cases	Several *T. equi, B. caballi*
Croatia	1 (*B. divergens*)	Endemic: *B. canis* (dominant)*, B. vogeli, B. gibsoni, B. vulpes* Sporadic cases: *B. caballi, T. equi, T. capreoli,*	nd	Endemic historically	Endemic: *T. equi, B. caballi*
Serbia	nd	Endemic: *B. canis* (dominant)*, B. vogeli, B. gibsoni, B. vulpes* Sporadic cases: *B. caballi, B. microti*	nd	Endemic historically, recently *Theileria* spp.	Endemic: *T. equi, B. caballi*
**Central Europe**					
Austria	8 cases, 3 published (2 *B. venatorum*, 1 *B. microti*) 5 unpublished (*B. venatorum, B. microti*) Seroprevalence 8%	*B. canis* endemic, common, mostly Eastern Austria (Dr endemic), *B. gibsoni* imported?	nd	Endemic, 1257 fatal cases *B. divergens* documented till 2 16, Alpine regions	One *T. equi* (autochthonous)
Czechia	2 cases (*B. microti)*	Recently *B. canis* endemic, SE region, Dr expanding 1x *B. gibsoni* imported	nd	Previously endemic No recent cases reported	*T. equi* and *B. caballi*
Germany	3 cases (2 *B. microti*, 1 *B. venatorum*) Seroprevalence up to 5 and 14%, for *B. divergens* and *B. microti*, respectively	*B. canis* endemic, increasing no. of autochthonous cases, Dr expanding 2x *B. gibsoni* - autochthonous	1, ‘exotic’ appearence	Endemic, recently one outbreak with high fatality rate due to *B. divergens*	Non endemic: But more *T. equi* and *B. caballi* pos horses reported
Hungary	1 case (*B. microti*)	*B. canis* endemic, common, Dr expanding,	nd	*B. divergens* used to be endemic,	*T. equi* endemic and *B.*

		32 *B. gibsoni* cases , 1 *B. vulpes* case<br>1 badger-associated Babesia sp. case		now extinct; *T. buffeli, T. orientalis* appeared	*caballi* only in Hortobágy
Luxembourg	nd	No cases but Dr newly settled in the South	1x *B. canis*-like	nd	nd
Poland	11 cases (9 *B. microti*)<br>1 *B. venatorum/B. divergens* infection asymptomatic<br>About 9% foresters and HIV-infected patients seropositive	*B. canis* endemic and expanding, Dr expanding<br>4x *B. gibsoni*	1x *B. canis*-like; sporadic cases (unpublished)	*B. divergens* endemic but no recent data published; Recent outbreak suspected	*T. equi* detected sporadically
Slovakia	3 cases (no data on species involved)	*B. canis* endemic and expanding, Dr expanding<br>2 *B. gibsoni* cases	nd	*B. divergens* endemic but no recent data published	nd
Slovenia	6 cases (5 *B. divergens* antibodies, 1 *B. crassa*-like)	*B. canis* endemic and expanding, Dr expanding<br>3 *B. vogeli* cases	nd	*B. divergens* endemic but no recent data published	nd
Switzerland	3 cases (2 *B. microti*, 1 *B. divergens)* regional seroprevalence of 1.5% for *B. microti*	*B. canis* endemic, low numbers (sporadic outbreaks), regional occurrence	*Cytauxzoon* spp in kittens	*B. divergens* endemic, sporadic cases (enzootic stability), *B. major* present, 1 outbreak *B. bigemina* (imported)	*T. equi* and *B. caballi* endemic, sporadic cases
**Northern and NE Europe**					
Denmark	1 case (*B. microti)*	1 *B. canis* autochthonous case, 1 imported. Dr introduced	nd	*B. divergens* endemic	nd

Estonia	nd	Sporadic *B. canis* cases, likely imported	nd	*B. divergens* endemic	EP (no species specified) diagnosed in horses in 2020
Finland	1 case (*B. divergens*)	Sporadic *B. canis* cases, imported	nd	*B. divergens* endemic	EP (no species specified) diagnosed in one imported horse in 2020
Iceland	nd	nd	nd	nd	nd
Latvia	nd	Recently *B. canis* endemic, SW region, Dr expanding	nd	nd	nd
Lithuania	nd	Recently *B. canis* endemic, expanding, Dr expanding	nd	nd	nd
Norway	1 case (*B. divergens*), antibodies to *B. microti* in 2.1% samples	1 likely autochthonous *B. canis* case, sporadic imported *B. canis*	nd	*B. divergens* endemic	nd
Sweden	2 cases (1 *B. divergens,* 1 *B. venatorum*) Antibodies to *Babesia* spp. in 4-16% samples	Imported cases *B. canis, B. gibsoni*	nd	*B. divergens* endemic	nd
**Summary**	**Human *babesiosis* in 13/20 countries Total 43 cases; *B. microti* in 7/20 countries *B. divergens* in 6/20 countries; *B. venatorum* in 3/20 countries**	**Endemic *B. canis* cases in 15/20 countries; *B. gibsoni* found in 9/20 countries (endemic in six); *B. vogeli* endemic in 3 Southern countries**	**3 cases of babesiosis published, in 3/20 countries**	***B. divergens* reported as endemic or previously endemic in 16/20 countries**	***T. equi* detected in 9/20 countries; *B. caballi* detected in 7/20 countries**

Nd – no data or not detected

Dr- *Dermacentor reticulatus*

EP- equine piroplasmosis

References are listed in the article.
